# Click DNA ligation with deoxyribozyme

**DOI:** 10.1093/nar/gkaf991

**Published:** 2025-10-02

**Authors:** Yangyang Chang, Yu Liang, Haodong Song, Qiang Zhang, Hong Yuan, Jiuxing Li, Zijie Zhang, Meng Liu

**Affiliations:** Central Hospital of Dalian University of Technology, Dalian University of Technology, Dalian 116000, China; School of Environmental Science and Technology, Dalian POCT laboratory, Key Laboratory of Industrial Ecology and Environmental Engineering (Ministry of Education), Dalian University of Technology, Dalian 116024, China; School of Environmental Science and Technology, Dalian POCT laboratory, Key Laboratory of Industrial Ecology and Environmental Engineering (Ministry of Education), Dalian University of Technology, Dalian 116024, China; School of Environmental Science and Technology, Dalian POCT laboratory, Key Laboratory of Industrial Ecology and Environmental Engineering (Ministry of Education), Dalian University of Technology, Dalian 116024, China; School of Bioengineering, Key Laboratory of Bio-Intelligent Manufacturing (Ministry of Education), Dalian University of Technology, Dalian 116024, China; Central Hospital of Dalian University of Technology, Dalian University of Technology, Dalian 116000, China; Central Hospital of Dalian University of Technology, Dalian University of Technology, Dalian 116000, China; School of Environmental Science and Technology, Dalian POCT laboratory, Key Laboratory of Industrial Ecology and Environmental Engineering (Ministry of Education), Dalian University of Technology, Dalian 116024, China; Central Hospital of Dalian University of Technology, Dalian University of Technology, Dalian 116000, China; School of Environmental Science and Technology, Dalian POCT laboratory, Key Laboratory of Industrial Ecology and Environmental Engineering (Ministry of Education), Dalian University of Technology, Dalian 116024, China; Central Hospital of Dalian University of Technology, Dalian University of Technology, Dalian 116000, China; School of Environmental Science and Technology, Dalian POCT laboratory, Key Laboratory of Industrial Ecology and Environmental Engineering (Ministry of Education), Dalian University of Technology, Dalian 116024, China

## Abstract

Deoxyribozymes that ligate DNA will expand the reaction scope of DNA catalysis and are useful in the construction of DNA nanostructures. Herein, we report the first efforts to isolate a novel class of DNA ligase deoxyribozymes from a random sequence DNA pool by *in vitro* selection. The identified deoxyribozymes catalyze the intermolecular linear DNA-DNA ligation via the formation of unnatural triazole linkages between a 5′ alkyne and a 3′ azide. One remarkable click-ligating deoxyribozyme, named CLDz2, ligated DNA with an observed rate constant (*k_obs_*) up to 2.7 × 10^−2^ h^−1^ at 10 mM Mn^2+^ (pH 7.0, 30°C), with up to 40% yield in overnight incubations. CLDz2 is predicated to have a four-way, junction-like structure comprised of four short duplexes, three hairpin loops, and two main interhelical unpaired elements. Comprehensive nucleotide covariation experiments suggest that CLDz2 should be generally applicable for click ligation of a wide range of 3′ azide DNAs. We further demonstrate a CLDz2-directed chemical ligation strategy for the synthesis of single-stranded monomeric circular DNA in high selectivity (97%), which can be used as a DNA template in rolling circle amplification.

## Graphical Abstract

**Graphical Abstract:**
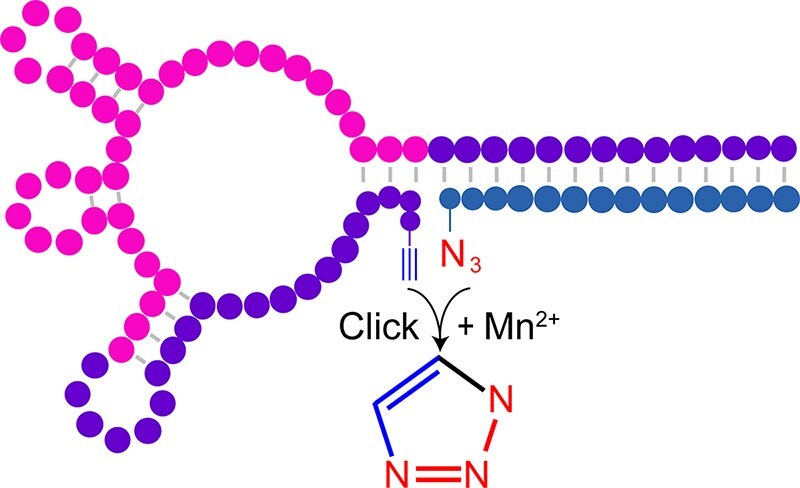

## Introduction

Deoxyribozymes (also referred to as catalytic DNAs, DNA enzymes or DNAzymes) are chemically synthetic, single-stranded DNAs capable of catalyzing chemical reactions [1–3]. Since the first deoxyribozyme was reported in 1994 for cleaving RNA, numerous deoxyribozymes have been identified by *in vitro* selection from random-sequence DNA pools [4–10]. Covalent nucleic acids ligation plays important roles in biochemistry, biotechnology and nanotechnology [11–14]. Ongoing efforts aimed at identifying deoxyribozymes that catalyze RNA ligation reactions in one of two ways: (i) joining a 2′,3′-cyclic phosphate with a 5′-hydroxyl, forming either native 3′-5′ or non-native 2′-5′ linear phosphodiester bonds [15–17]; (ii) linking a 2′-(or 3′)-hydroxyl with a 5′-triphosphate, forming either non-native 2′-5′ linear RNA, native 3′-5′ linear RNA, or 2′,5′-branched RNA [18–20].

Beyond RNA ligation, two classes of DNA-ligating deoxyribozymes (DLDz) have been identified for catalysis of DNA intermolecular ligation with (i) 3′-phosphorimidazolide and 5′-hydroxyl [21], and (ii) 3′-hydroxyl and 5′,5′-pyrophosphate linkage, resulting in the formation of a new 3′,5′-phosphodiester linkage [22]. Among the possible ligation products, linear nucleic acids (LNAs) with a native 3′-5′ linkage is particularly interesting and useful in synthesizing large nucleic acid polymers from smaller fragments. However, DLDz were and still are rare. Furthermore, the currently known DLDz have stringent sequence requirements and reaction conditions. The first DLDz named E47 required either Zn^2+^ or Cu^2+^ to join a 5′-hydroxy with a 3′-phosphorimidazolide. In addition, the sequence tolerance of E47 was not clear. In the latter example, the DLDz named L115 catalyzed the formation of a 3′,5′-pyrophosphate linkage between acceptor and donor DNA molecules. However, the particular sequence requirement of the donor DNA substrate limits the range of sequences that can be ligated. Although DLDz are at their infancy, recent studies demonstrated their great promise in bioanalysis, biosensor, and DNA machines [23].

Encouraged by a study conducted by Yu and Sen in which a deoxyribozyme (named CLICK-17) was isolated to catalyze the alkyne-azide click (AAC) reaction in the presence of sub-micromolar Cu^+^ [24], we herein carried out an *in vitro* selection to derive deoxyribozymes from a random-sequence DNA pool that could perform copper-free click DNA ligation. Furthermore, this AAC reaction was chosen for several practical reasons: (i) alkynes and azides can be readily attached to DNA strands without disturbing their biophysical properties; (ii) the resulting triazole linkage is chemically stable and biologically compatible; (iii) it has facilitated the construction of various chemically modified DNA oligonucleotides in biology and nanotechnology [11–14, 25]. The classical click reaction, known as the copper-catalyzed AAC (CuAAC), relies on a Cu(I) catalyst to facilitate the reaction of a terminal azide with an alkyne to form a 1,4-disubstituted 1,2,3-triazole linkage [26–28]. In this study, we reported click-ligating deoxyribozymes (CLDz) that catalyzed the AAC for DNA ligation *via* the formation of unnatural triazole linkages. The most intriguing finding is that CLDz used Mn^2+^ as the metal ion cofactor for the classical AAC. The identified CLDz were found to ligate DNAs with the observed rate constant (*k_obs_*) up to 2.7 × 10^−2^ h^−1^ at 10 mM Mn^2+^ (pH 7.0, 30°C), and with up to 40% ligation yield following 24 h incubation. We envisioned that this work would broaden the scope of nucleic acid ligation known to be catalyzed by DNA, and expand the applications of DNA ligase deoxyribozymes.

## Material and methods

### Materials and reagents

#### Oligonucleotides and materials

The DNA oligonucleotides listed in Supplementary Table S1 were obtained from Sangon Biological Engineering Technology & Services Co., Ltd. (Shanghai, China) and Generay Biotech Co. Ltd. (Shanghai, China). An NHS ester functional group was used to attach an azide moiety at the 3′ end of acceptor DNA (AD1). And an alkynyl was attached to the 5′ end of DNA pool (DP1) *via* the phosphoramidite bond. All these purchased oligo sequences were purified by high-performance liquid chromatograph (HPLC) or 10% denaturing polyacrylamide gel electrophoresis (dPAGE, 8 M urea) before use. Potassium chloride (KCl) was purchased from Sangon Biotech Co., Ltd. (Shanghai, China). 4-hydroxyethyl piperazine ethanesulfonic acid (HEPES, ≥ 99%), magnesium chloride hydrate (MgCl_2_·6H_2_O, ≥ 98%), urea, Tris-HCl, 40% polyacrylamide solution (29:1) and EDTA-2Na were acquired from Solarbio Science & Technology Co., Ltd. (Beijing, China). DNase I and DNase/RNase-free water were acquired from Thermo Fisher Scientific Inc. (Waltham, USA). Tween-20 was obtained from BBI Life Sciences Corporation (Shanghai, China). Tris(3-hydroxypropyltriazolylmethyl)amine (THPTA) was purchased from Xi’an Ruixi Biotech Co., Ltd (Xi’an, China). All other chemicals were bought from Shanghai Aladdin Bio-Chem Technology Co., LTD. (Shanghai, China) and used without further purification.

#### Instruments

The fluorescent images of gels were obtained using a Typhoon 5 variable mode imager (GE Healthcare, US) and analyzed using Image Quant software (Molecular Dynamics). The ligated product was digested by DNase I and determined using high-performance liquid chromatography/electrospray ionization quadrupole time-of-flight mass spectrometry (HPLC-ESI-QTOF/MS, Agilent G6546A, USA). The ssDNA concentration was quantified by NanoDrop One (Thermo Scientific, USA). All of polymerase chain reactions (PCR) were performed using MyGo Pro (IT-IS Life Science, UK) and T100™ PCR Cycler (Bio-Rad, USA).

#### Buffers used in this work

2 × Selection buffer a (SB-a): 50 mM HEPES, 300 mM NaCl, 40 mM KCl, 40 mM MgCl_2_·6H_2_O and 0.02% Tween 20 (pH 7.5); 2 × Selection buffer b (SB-b): 50 mM HEPES, 0.02% Tween 20 (pH 7.5); 2 × Selection buffer c (SB-c): 50 mM HEPES, 0.02% Tween 20 (pH 7.0); 10 × PCR buffer: 100 mM Tris-HCl, 15 mM MgCl_2_, 500 mM KCl (pH 8.9); 10 × Reaction buffer (RB): 100 mM Tris-HCl, 25 mM MgCl_2_, 1 mM CaCl_2_ (pH 7.5 at 25 °C).

### In vitro selection procedures

#### DP1/AD1 duplex assembly (step i)

600 pmol AD1 and 300 pmol DP1 were mixed in 50 μL of 2 × SB-a, and then diluted to 90 μL by adding ddH_2_O. The mixture was incubated at 90°C for 5 min, and then cooled at room temperature (RT) for 10 min.

#### Click-ligation of AD1 to DP1 (step ii)

10 μL of MnCl_2_ (200 mM) was added into the above mixture and incubated at 30°C for 8 h. The reaction was stopped by ethanol precipitation.

#### Purification of click-ligated DP1-AD1 products (step iii)

The obtained product from step ii were separated and purified by 10% dPAGE. The purified ligated product was dissolved into 20 μL of ddH_2_O and stored at −20°C before use. The amount of ligated product was quantified using Image Quant software. And the ligation percentage (Lig%) was calculated by the following equation (1):


(1) \begin{eqnarray*}
{\mathrm{Lig\% = }}\left( {{\mathrm{100*lig}}} \right){\mathrm{/}}\left( {{\mathrm{lig + un - lig}}} \right)
\end{eqnarray*}

where lig and un-lig represent the band intensities of ligated and un-ligated products, respectively.

#### Standard PCR1 (step iv)

Around 1 μL of ligated product (PCR1 template), 0.5 μL of forward primer FP1 (100 μM), 0.5 μL of reverse primer RP1 (100 μM), 5 μL of 10 × PCR buffer, 1.2 μL of dNTPs (dATP, dCTP, dGTP and dTTP, 2.5 mM), and 1 μL of Thermus thermophilus DNA polymerase (5 U/μL) were mixed and diluted to 50 μL using ddH_2_O. The target DNA was amplified using the following steps: 94°C for 1 min; 10–15 cycles of 94 °C for 30 s, 50 °C for 45 s and 72 °C for 40 s; 72 °C for 5 min.

#### PCR2 (step v)

Around 5 μL of the PCR1 product was diluted with ddH_2_O to 100 μL, which was acted as the template for PCR2. Around 1 μL of PCR2 template was used for amplification based on the same protocol with PCR1 except that RP1 was replaced by reverse primer RP2. To achieve full amplification, the cycle numbers among different selection rounds were adjusted, generally between 11 to 13 cycles, according to the RT-PCR results.

#### Purification of deoxyribozyme-coding strand by dPAGE (step vi)

The existence of C3 spacer in RP2 prevents the amplification of the A21 fragment, the antisense strands are longer than the sense strands, which is prone to distinguish the sense strands from the PCR2 products. The obtained sense strands were purified by 10% dPAGE and used for the next round of selection.

Ten additional rounds of selection were performed as described above except the DP1 amount was reduced to 200 pmol at rounds 2–3, 100 pmol at rounds 4–7, 80 pmol at round 8, 50 pmol at round 9, 40 pmol at round 10 and 30 pmol at round 11; and the positive-selection time was reduced to 4 h at rounds 7–8, and 2 h at rounds 9–11. The DNA population after 11 rounds of *in vitro* selection was subjected for deep sequencing using the MiSeq (Illumina) sequencing platform.

### Digestion of the ligated product

Around 20 μL of the purified ligated product (100 μM), 60 μL of DNase I (1 U/μL) and 20 μL of 10 × RB were mixed and diluted to 200 μL using ddH_2_O. After incubation at 37°C for 60 min, the reaction was stopped by adding 10 μL of 50 mM EDTA. The obtained mixture was then transferred into a 10 KD ultrafiltration tube for centrifugation at 8000 rpm for 20 min. The filtrate was subjected for mass spectrographic analysis.

### Determination of the digestion product

The digestion product was analyzed by an Agilent HPLC-ESI-QTOF/MS. We injected 100 μL of sample into the LC system at a flow rate of 0.3 mL/min. The analyte was separated using a XB-C18 chromatography column (3 mm x 100 mm) at 30°C. The mobile phase used for gradient elution consists of (A) 0.1% formic acid aqueous solution and (B) methanol. The gradient elution condition was: 0 min 95% A, 3 min 75% A, 6 min 75% A, and 15 min 95% A. Data was obtained using negative ion mode (ESI-) over mass spectrometry range of m/z 100–1100. Full scan was recorded with a resolution of 60 000 FWHM. The source parameters were set as follows: gas temperature: 300°C; gas flow: 8 L/min; nebulizer pressure: 35 psig; Nozzle voltage: 1000 V. The mass spectrometer was configured to isolate and monitor the protonated molecules [M + HCOO]^−^ at m/z 481.20.

### Divalent metal ion dependency analysis of CLDz2.

Around 6 μL of CDLz2 (10 μM), 2 μL of AD1 (10 μM) and 50 μL of 2 × SB-b were mixed and diluted to 90 μL using ddH_2_O. After incubation at 90°C for 5 min, the mixture was cooled at RT for 10 min. Around 10 μL of different divalent metal ion (200 mM), including Mg^2+^, Mn^2+^, Co^2+^, Ni^2+^, Pb^2+^, Ca^2+^, Zn^2+^, Ba^2+^ and Cu^2+^, was, respectively, added into the above mixture and incubated at 30°C for 24 h. Followed by ethanol precipitation, the resultant products were analyzed by 10% dPAGE.

### Kinetic analysis of CLDz2 and CLSS1

Kinetic analysis of CLDz2 to ligate AD1 was performed as follows: 6 μL of CLDz2 (10 μM), 2 μL of AD1 (10 μM) and 50 μL of 2 × SB-c were mixed and diluted to 95 μL using ddH_2_O. After incubation at 90°C for 5 min, the mixture was cooled at RT for 10 min. 5 μL of 200 mM MnCl_2_ was added into the above mixture. After incubation at 30°C for 4, 8, 12, 18, 24, 36, 48, 60, and 72 h, the reaction was stopped by ethanol precipitation. The resultant products were analyzed by 10% dPAGE. Kinetic analysis of CLSS1 was carried out as described above except for: CLDz2 was replaced by CLSS1. Percent ligation of CLDz2 or CLSS1 versus reaction time was plotted and non-linear fitted.

### Reselection and DNA alignment

DP1 was mutated with a 45% mutation rate and the Mn^2+^ concentration was reduced to 10 mM. The reselection protocol was similar to the one described in “In vitro selection procedures,” except for: (1) the DNA pool amount was reduced to 300 pmol at round 2, 150 pmol at round 3, 75 pmol at round 4, and 50 pmol at rounds 5–7. (2) the positive-selection time was, reduced to 6 h at round 5, 3 h at round 6, and 1.5 h at round 7. The DNA population after seven rounds of *in vitro* selection was subjected for deep sequencing using the MiSeq (Illumina) sequencing platform. We aligned the most populated 41639 sequences, that belong to 508 families, to the reference sequence (CLDz2) for calculating variation index (VI) based on the following equation (2):


(2) \begin{eqnarray*}
{\mathrm{VI = }}\left[ {{\mathrm{1 - }}\left( {{\mathrm{N/Nt}}} \right)} \right]{\mathrm{/0}}{\mathrm{.45}}
\end{eqnarray*}

where N represents the amounts of sequences that have the same base with reference sequence at the same position, and N_t_ represents the total amounts of sequences, 0.45 is the mutation rate at each position.

### Intramolecular circularization of linear DNA (LDNA)

Kinetic analysis of LDNA1 (Supplementary Table S1) for circularization was carried out using the similar protocol as described in Section “Kinetic analysis of CLDz2 and CLSS1” except that: (1) 5 μL of LDNA1 (10 μM) was used instead of CLDz2 and AD1; (2) the reaction time was set at 1, 2, 4, 8, 12, and 24 h. The circularization product (CDNA1) was analyzed using 10% dPAGE. The circularization yield (Y)% was defined as 100 × CP/(un-lig + CP + LLP); the circularization selectivity (S%) was defined as 100 × CP/(CP + LLP), where un-lig, CP and LLP represent band intensities of un-ligated product, circularization product and linear ligated product, respectively.

### Rolling circle amplification (RCA)

Around 1 μL of CDNA1 (1 μM), 5 μL of dNTPs (10 mM), 1 μL of RCA primer (5 μM, Supplementary Table S1), 5 μL of 10 × RCA buffer, and 1 μL of phi29DP (10 U/μL) were mixed and diluted to 50 μL using DNase/RNase-free water. The reaction was performed at 30°C for 0, 6, 12, 16, 20, 24, and 36 h before heating at 65°C for 10 min. The RCA products from these reactions were analyzed by 0.6% agarose gel electrophoresis.

## Results

### In vitro selection of click-ligating deoxyribozymes (CLDz)

**Figure 1.**
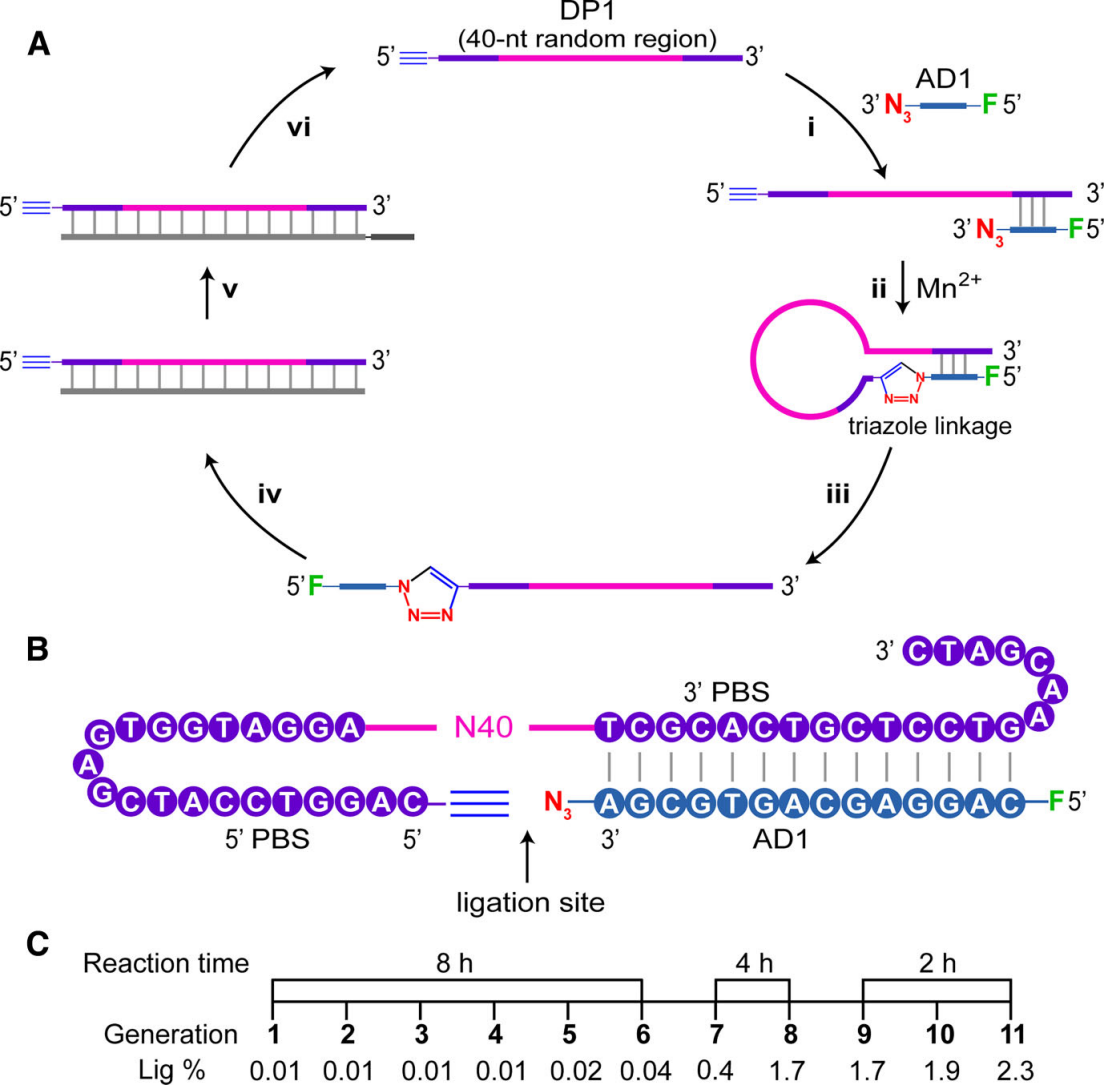
Selection of click-ligating deoxyribozymes (CLDz). (**A**) *In vitro* selection scheme. DP1: DNA pool with 40 random nucleotides (N_40_); AD1: acceptor DNA. Each selection cycle consists of steps i-iv. (i) Formation of the DP1/AD1 duplex assembly; (ii) Click-ligation of AD1 to DP1; iii) Purification of click-ligated DP1-AD1 products; (iv) Standard PCR1; (v) PCR2 using reverse primer containing a C3 spacer and A21 tail; (vi) Purification of CLDz-coding strand by dPAGE. (**B**) The sequences of DP1 and AD1 used to assemble a duplex structure. PBS: primer binding sequence. (**C**) *In vitro* selection progress. The reaction time and ligation percentage (Lig%) for each selection cycle are indicated.

To isolate catalytic DNAs with click-ligation activity (named CLDz), we employed an *in vitro* selection strategy depicted in Fig. 1A. The starting DNA pool, denoted DP1, contained, in the 5′ to 3′ direction, a 5′ alkyne group, a 5′ primer-binding site (5′ PBS, 21-nt, nt: nucleotide), a 40-nt random domain (N_40_), and a 3′ primer-binding site (3′ PBS, 21-nt). An acceptor DNA (AD1, 14-nt) was labeled with a fluorescein (F) and an azide at its 5′ and 3′ end, respectively (see Supplementary Table S1 for all DNA molecules used in this study). The chemical structures of the azide and alkyne groups attached to the DNA were shown in Supplementary Fig. S1. In step i, DP1 (∼ 10^14^ molecules) was incubated with AD1 to assemble into the bipartite duplex structure (Fig. 1B) in 1 × selection buffer a: 25 mM HEPES, 150 mM NaCl, 20 mM KCl, 20 mM MgCl_2_·6H_2_O and 0.01% Tween 20 (pH 7.5). Click-ligation reactions between the 5′ alkyne of DP1 and the 3′ azide group of AD1 was carried out in the presence of 20 mM Mn^2+^ at 30°C (step ii). This step was aimed to produce a click-ligated product (CLP) containing a single triazole linkage at the ligation site. The resultant CLPs were isolated by denaturing PAGE (dPAGE, step iii), and amplified by two PCRs (PCR1 and PCR2). PCR1 used two standard primers, forward primer FP1 and reverse primer RP1 (step iv). PCR2 used FP1 and RP2 (step v). Since RP2 contained a C3 spacer that prevents the amplification of its A21 tail, the coding strand of the DNA product can be easily separated from the longer non-coding strand by dPAGE (step vi). Such purified DNA pool was then used for the next round of selection. A total of 11 cycles of selection were conducted. The DNA population from round 11 was subjected to deep sequencing.

The selection progress was monitored through the ligation percentage (Lig%) of the DNA pool in each round of selection (Fig. 1C). The click-ligation reaction was first allowed to proceed for 8 h in G1-G6, and the reaction time was reduced to 4 h in G7-G8, and finally to 2 h in G9-G11 to isolate the most efficient deoxyribozymes. No obvious click-ligation activity was observed for the DNA population in generations G1-G6. However, more than 2% of the population generated was ligated to AD1 after 11 rounds of selection (G11).

The click-ligating activity of the top three ranking sequences was tested (Supplementary Fig. S2, Supplementary Table S2). The rank 2 sequence demonstrated the highest ligation activity (27%) after 24-h incubation at 30°C. This sequence was named CLDz2 and chosen for further investigation.

### Characterization of CLDz2

**Figure 2.**
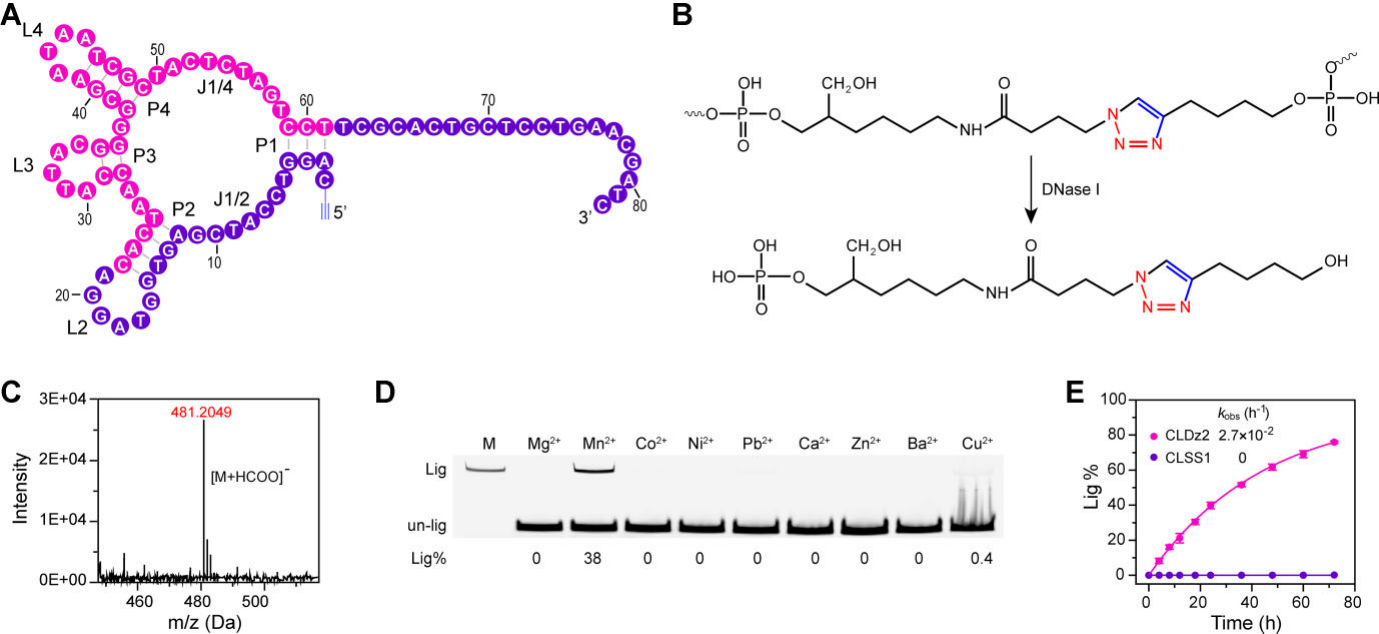
Characterization of CLDz2. (**A**) A secondary structure of CLDz2 predicted by the M-fold program. P, a base-paired region; L, a loop region; J, a junction between two base-paired regions. Random-sequence nucleotides are colored in pink. Primer-binding nucleotides are colored in purple. (**B**) Chemical structures at ligation point before and after DNase I treatment. (**C**) Exact mass spectrum of the digestion mixture with annotation of the proposed 1,2,3-triazole product peaks. (**D**) 10% dPAGE analysis of the click-ligating activity of CLDz2 in the presence of different divalent metal ions. Reaction time: 24 h, lig: ligated product, un-lig: un-ligated product, Lig%: ligation percentage, M: marker. (**E**) Kinetic profile of the click-ligation reaction of CLDz2 and CLSS1.

Fig. 2A shows the predicted secondary structure of CLDz2, which contains four short duplexes (P1-P4), three hairpin loops (L2-L4), and two main interhelical unpaired elements (J1/2 and J1/4). To confirm the click ligation of azide AD1 to alkyne CLDz2 to yield a DNA strand containing a single triazole at the ligation site, the purified CLPs were subjected to complete digestion with DNase I. The chemical structure of the resultant 1,2,3-triazole linkage is shown in Fig. 2B, as confirmed by HPLC-ESI-QTOF/MS [M + HCOO^−^ calc, 481; found, 481] (Fig. 2C).

Metal ions play important roles in the catalytic activity of deoxyribozymes. CLDz2 was first assessed for divalent metal dependences. CLDz2 was extremely specific for Mn^2+^, but was inactive in the presence of only Mg^2+^, Co^2+^, Ni^2+^, Pb^2+^, Ca^2+^, Zn^2+^, or Ba^2+^ (Fig. 2D). It is noteworthy that CLDz2 was incapable of performing catalysis in the presence of Cu^2+^. However, CLDz2 can perform the DNA-templated chemical ligation in the presence of as little as 5 μM Cu^+^ (Supplementary Fig. S3). Furthermore, CLDz2 had no activity when monovalent metal ions (i.e. K^+^ and Na^+^) were used. In contrast, the presence of K^+^ and Na^+^ even slightly reduced the activity of CLDz2 (Supplementary Fig. S4). The effect of Mn^2+^ concentration on the activity was also tested, and it was found that the Lig% increased with increasing Mn^2+^ concentration, with a Lig% of 37% at 10 mM Mn^2+^ (Supplementary Fig. S5). We also examined the catalytic activity of CLDz2 under different reaction pH and temperatures. A robust click-ligating activity was observed at pH 7.0–7.5 (Supplementary Fig. S6) and 30°C (Supplementary Fig. S7). It is not surprising since the original deoxyribozyme was isolated at pH 7.5 at 30°C. Further characterization of CLDz2 was conducted under the following reaction conditions: 25 mM HEPES (pH 7.0 at 23°C) containing 10 mM MnCl_2_ at 30°C.

The CLDz2 construct was then used to establish the kinetic characteristics of deoxyribozyme-mediated click DNA ligation (Supplementary Fig. S8). We determined that the full-length CLDz2 has an observed rate constant (*k_obs_*) of 2.7 × 10^−2^ h^−1^ and a maximum ligation yield (Y_max_) of 76% in 72-h incubations (Fig. 2E). In contrast, a scrambled sequence, CLSS1 (Supplementary Table S1), was tested as a control. No obvious ligation product was observed for CLSS1 (Supplementary Fig. S9), indicating that the click-ligating activity by the deoxyribozyme is sequence-specific. Note that the maximum *k_obs_* value under single-turnover conditions for CLDz2 was comparable or even higher than those for the previously reported deoxyribozymes E47^21^ and L115^22^ under neutral condition (Supplementary Table S3).

### Sequence optimization by nucleotide truncation.

**Figure 3.**
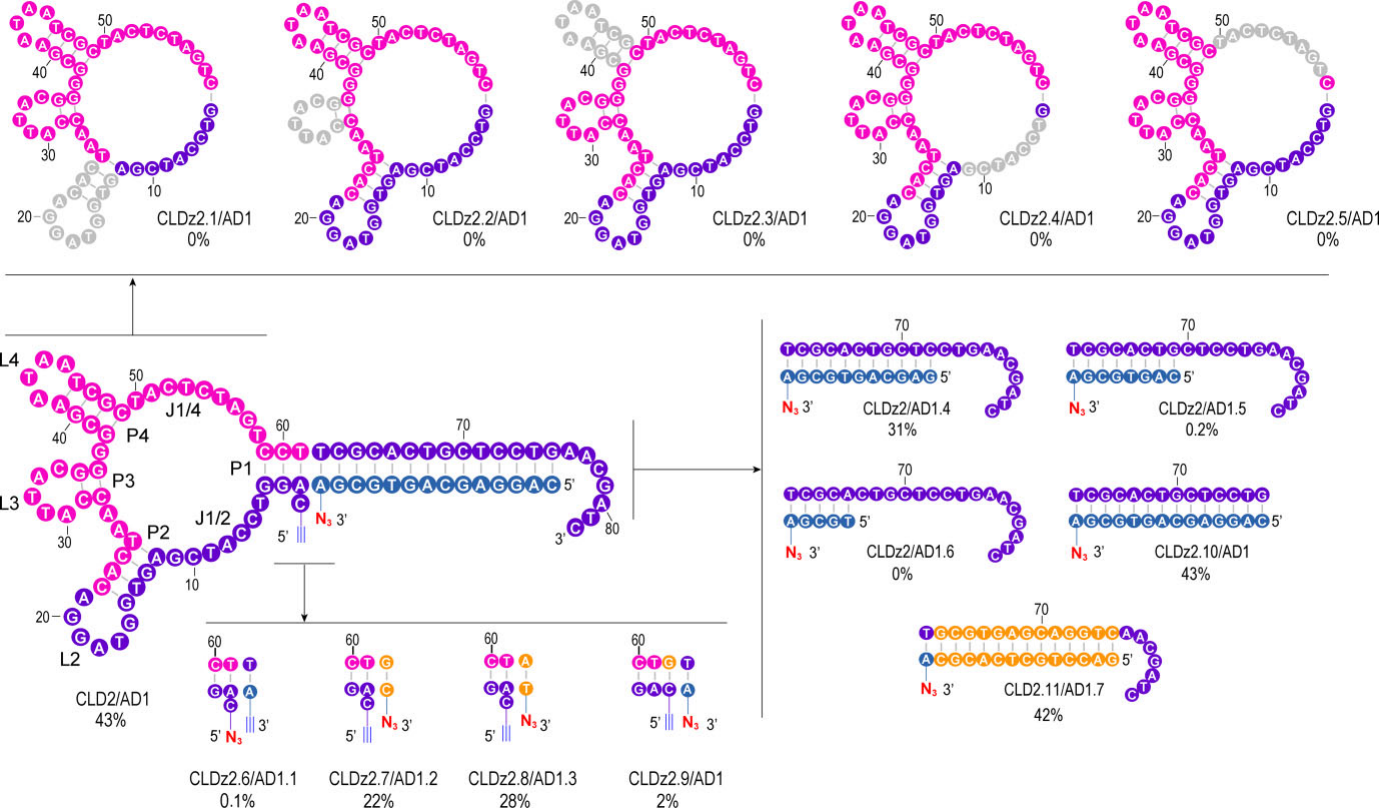
Sequence truncation of CLDz2. Nucleotides shown in gray circles are the removed nucleotides in each construct in comparison to the wild-type CLDz2, and in yellow circles are the actual altered nucleotides. Lig% is shown below each construct.

We next examined the functionally essential nucleotides within CLDz2 (Fig. 3). CLDz2 variants in which L2 + P2 (CLDz2.1), L3 + P3 (CLDz2.2), L4 + P4 (CLDz2.3), J1/2 (CLDz2.4), or J1/4 (CLDz2.5) were removed and exhibited no click-ligating activity (Lig%: 0%) (Supplementary Fig. S10). This result indicated that these sequence elements are crucial to the deoxyribozyme function.

We sought to investigate the effects of nucleotides variations at the ligation site on the click-ligation activity. We first examined whether the position of 5′ alkyne and 3′ azide present on CLDz2/AD1 complex affected the activity. The original alkyne and azide modifications are inverted, producing a 5′ azide-modified CLDz2.6 and a 3′ alkyne-labeled AD1.1. This resulted in the loss of activity for CLDz2.6/AD1.1 construct (Lig%: 0.1%, 24 h) (Supplementary Fig. S11). Three more variants were also tested. Changing the initial T62-A pairs (within CLDz2/AD1) to G62-C pairs (within CLDz2.7/AD1.2), or A62-T pairs (within CLDz2.8/AD1.3) caused noticeable reductions in Lig% (43% versus 22%; 43% versus 28%). Enlarging CLDz2 by one additional base pair (G62-C) at the 5′ end caused a very significant loss of activity for CLDz2.9 (Lig%: 2%) (Supplementary Fig. S12).

We also examined the required number of base pairs (bp) in the standard duplexes of CLDz2/AD1. Three shortened acceptor DNA molecules (AD1.4, AD1.5, and AD1.6) were chemically synthesized and assessed (Supplementary Fig. S13). CLDz2/AD1.4 constructs with 11 bp were indeed active (Lig%: 31%). However, further shorting the binding length to 8 bp resulted in very significant loss of activity in CLDz2/AD1.5 (Lig%: 0.2%). The CLDz2/AD1.6 construct with only 5 bp became inactive (Lig%: 0%). Removal of overhang nucleotides (7 nt) at the 3′ end resulted in the CLDz2.10 variant with the retained activity (Lig%: 43%) (Supplementary Fig. S14).

To assess the importance of the duplex structure, we also made a construct, CLDz2.11/AD1.7 in which the base-pairing content was arbitrarily altered from the original CLDz2/AD1, while Watson–Crick interactions were maintained. This construct was fully active (Lig%: 42%) (Supplementary Fig. S13). Taken together, our data support the following conclusions: (1) the base-pair content of the duplex is not important; (2) a stable duplex structure is required for the optimal activity.

### Reselection with a mutagenized CLDz2 pool

**Figure 4.**
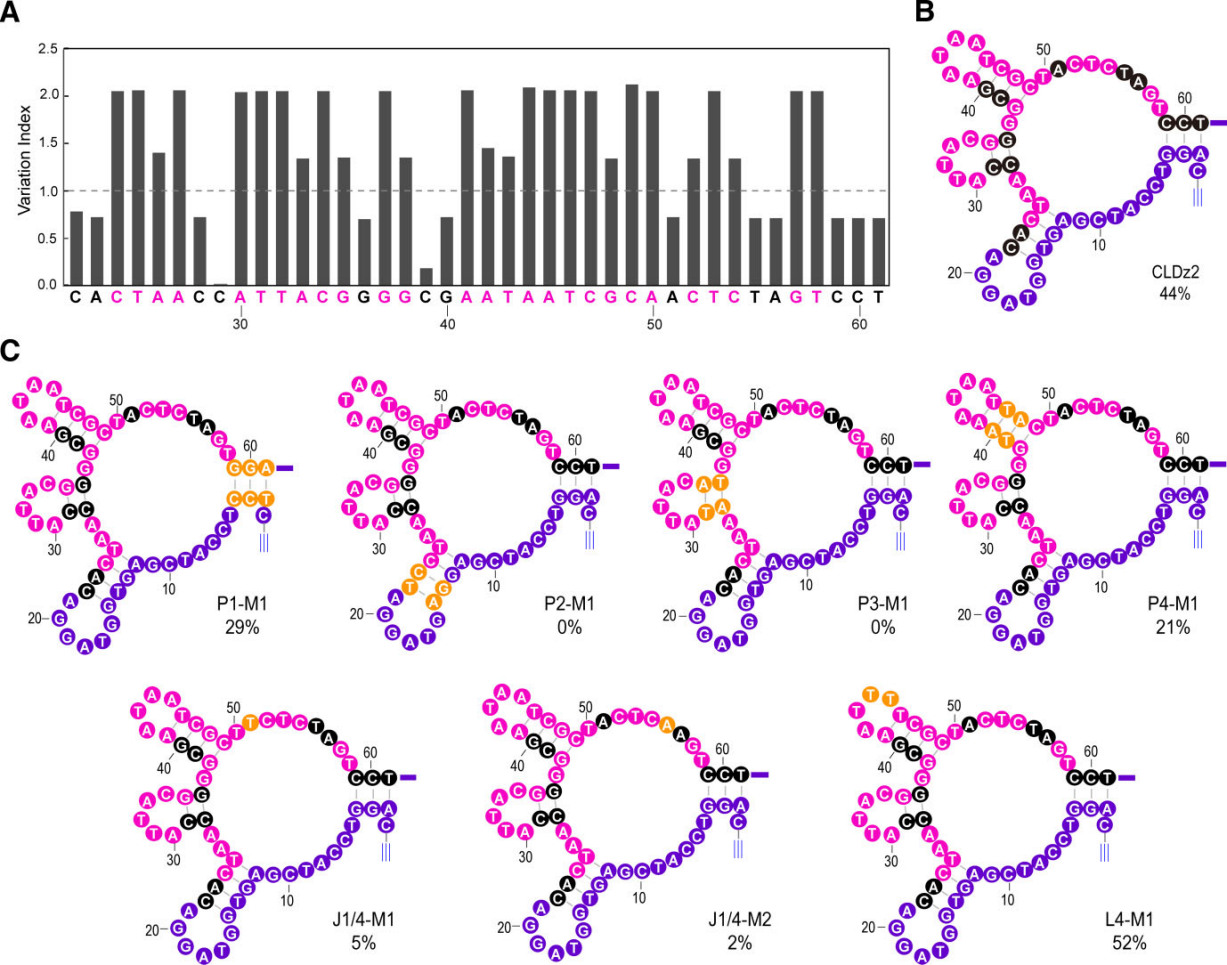
CLDz2 reselection. (**A**) Sequence analysis of the random-sequence pool from reselection. Black-coded nucleotides are functionally important (VI < 1). Pink-coded nucleotides tolerate some level of mutation (VI > 1). (**B**) The putative secondary structure of fully functional CLDz2 with all nucleotides properly colored. (**C**) Examination of functionally essential nucleotides of CLDz2. Nucleotides colored in yellow are the actual altered nucleotides compared to the wild-type CLDz2. Lig% is shown below each construct.

To further identify the nucleotides in CLDz2 that are catalytically essential, a reselection experiment was performed using a partially randomized library of CLDz2. This DNA library was chemically synthesized with a 45% mutation rate at each nucleotide within the original random-sequence domain. The *in vitro* selection scheme was identical to that used in the original isolation of CLDz2. The click-ligation reaction was reduced to 6 h in G5, to 3 h in G6, and finally to 1.5 h in G7 (Supplementary Fig. S15). The round-7 DNA pool was sequenced. The most populated 508 families were aligned against CLDz2 to identify conserved nucleotides (Supplementary Table S3). The original nucleotides of CLDz2 are shown with the variation index (VI, a measure of variability) (Fig. 4A). VI was calculated by dividing the observed mutation rate at each nucleotide by 45%. Nucleotides that were functionally important were colored in black (VI < 1). In contrast, nucleotides colored in pink can tolerate mutations (VI > 1). Fig. 4B indicated the important sequence element from reselection results, in accordance with that identified by the sequence truncation results.

We also designed and tested the activity of CLDz2 mutants to further verify the importance of nucleotides to the deoxyribozyme function (Fig. 4C and Supplementary Fig. S16). When A2-T61, G3-C60, and G4-C59 pairs of P1 were covaried to T2-A61, C3-G60, and C4-G59, the mutant construct (P1-M1) exhibited a decreased activity (29% versus 44%). For P2, changing T14-A23 and G15-C22 to G14-C23 and A15-T22 caused complete loss of activity (P2-M1). Similarly, when the two C-G pairs of P3 were covaried into two A-T pairs (P3-M1), the construct became inactive.

The validity of P4 was further tested by replacing the C-G pairs with A-T pairs (P4-M1). The enzymatic activity was decreased by half (21% versus 44%). For J1/4, mutating A51 to T51 (J1/4-M1) or T55 to A55 (J1/4-M2) significantly reduced the catalytic activity (5% and 2%). For L4, replacing A45 and A46 with T45 and T46 slightly enhanced the activity (52% for L4-M1).

**Figure 5.**
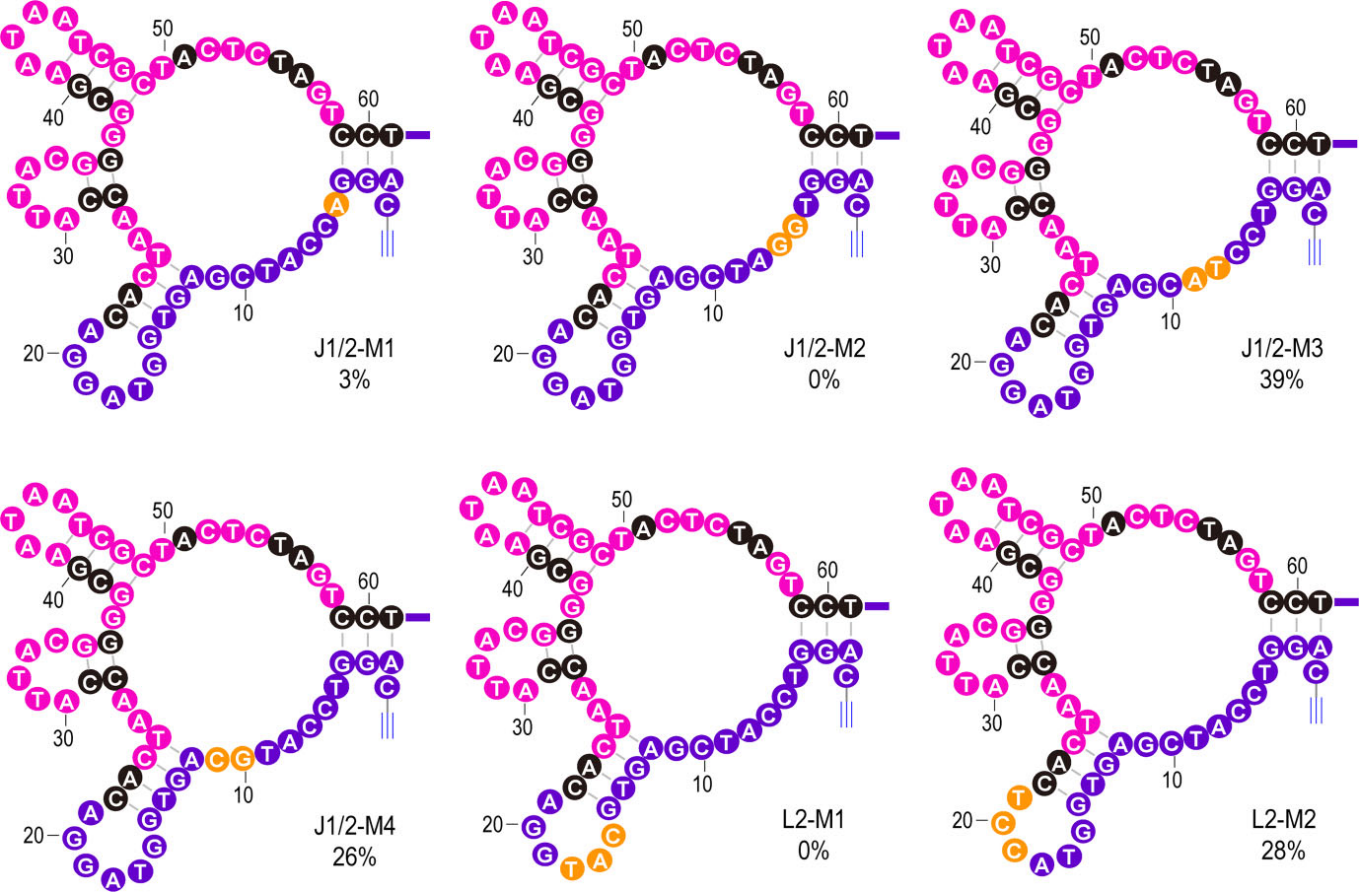
Examination of functionally essential nucleotides within L2 and J1/2 of CLDz2. Nucleotides colored in yellow are the actual altered nucleotides compared to the wild-type CLDz2. Lig% is shown below each construct.

We finally examined the importance of nucleotides in J1/2 and L2 within CLDz2 (Fig. 5 and Supplementary Fig. S17), which belong to 5′ PBS and are not mutated during reselection. For J1/2, when T5 was mutated to A5 (J1/2-M1), C6 and C7 to G6 and G7 (J1/2-M2), the corresponding Lig% were significantly reduced to 3% and 0%, respectively. In contrast, changing A8 and T9 to T8 and A9 (J1/2-M3) did not affect the activity. The mutant J1/2-M4, containing C10-to-G10 and G11-to-C11 mutations, exhibited a reduced catalytic activity (26%). Three nucleotides within L2, G16, T17, and A18, appear to play crucial roles in the catalytic function of CLDz2, because mutating these nucleotides (G16-to-C16, T17-to-A17, and A18-to-T18) resulted in the complete loss of activity in L2-M1. Further mutation of the remaining three nucleotides in L2 (G19-to-C19, G20-to-C20, and A21-to-T21) resulted in a L2-M2 construct that was still active but with a Lig% reduced from 40% to 28%.

### Synthesis of single-stranded circular DNA and rolling circle amplification

**Figure 6.**
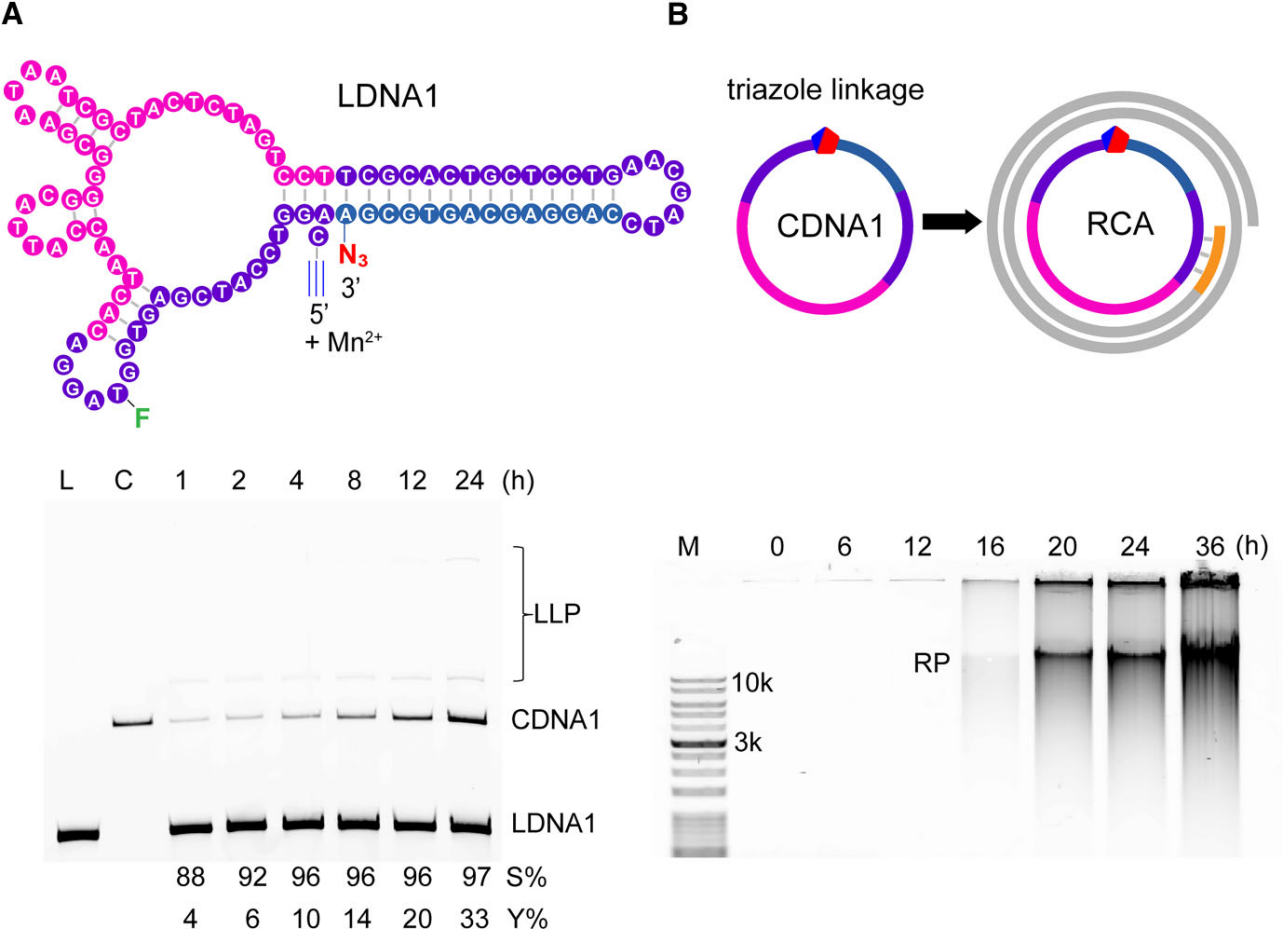
(**A**) Time-dependent circularization of LDNA1 in the presence of Mn^2+^. Lane L: LDNA1; Lane C: CDNA1; LLP: linear ligated products; Y%: circularization yield; S%: circularization selectivity. (**B**) Time-dependent RCA using CDNA1 as the template. M: marker; RP: RCA product.

Recently, circular nucleic acids (CNAs) have attracted extensive attention in the field of diseases diagnosis, drug delivery, biosensing, and DNA nanotechnology.[13, 14] Different from LNAs, CNAs are characterized by a closed-loop structure that are formed by intramolecular ligating of two ends of LNAs. Typically, the protein enzymes T4 DNA ligase, CircLigase or T4 RNA ligase are used for preparation of CNAs, but challenges with selectivity and yield are common, because the entropic difference between intramolecular circularization and intermolecular ligation is small [29, 30]. CLDz2 used defined sequence as the structural requirements to carry out click-ligation functions, we speculate that it may be possible to derive deoxyribozyme-directed chemical ligation methodology for exclusive production of single-stranded circular DNA (CDNA). A linear DNA (LDNA1, 96-nt) was designed to contain, in the 5′ to 3′ direction, the original CLDz2 sequence and the AD1 sequence. In the presence of Mn^2+^, LDNA1 with 5′ alkyne and 3′ azide functionalities, underwent an intramolecular click-ligation reaction, and formed a single-stranded CDNA1 construct. Fig. 6A showed that LDNA1 exhibited a yield of circularization (Y%) of 33% for the formation of CDNA1 in 24 h. Furthermore, we used the circularization selectivity (S%) to describe the selectivity of LDNA1 for intramolecular circularization over intermolecular polymerization. Remarkably, the monomeric CDNA1 was directly synthesized in high selectivity (S% = 97%).

Because the click-ligated CDNA1 contains an unnatural triazole linkage, we investigated its ability as a DNA template for rolling circle amplification (RCA) using phi29 DNA polymerase (phi29DP). Agarose gel analysis indicated that RCA products (RP) greater than 10 kb were produced after 16 h (Fig. 6B). The overall slow polymerization rate of the RCA reaction could be the result of the extended unnatural backbone at the site of click-ligated CDNA1.

## Discussion

We used *in vitro* selection to identify new deoxyribozymes that catalyzed the classical AAC using Mn^2+^ as the metal ion cofactor. One of these deoxyribozymes, CLDz2 has the ability to ligate DNAs *via* the formation of unnatural triazole linkages with an observed reaction rate constant (*k_obs_*) of 2.7 × 10^−2^ h^−1^ at 10 mM Mn^2+^ (pH 7.0, 30°C). This represents a fundamentally new DNA-catalyzed reaction, thus expanding the repertoire of chemical reactions known to be catalyzed by DNA. For comparison, CLICK-17 catalyzed conjugation between alkyne and azide substrates with sub-micromolar Cu^+^ or Cu^2+^ as added cofactors [24]. Therefore, CLDz2 should be highly biocompatible in future biological applications. Since Mn^2+^ was not able to catalyze the AAC reaction, the most intriguing result is that CLDz2 is catalytic with Mn^2+^ as added cofactor. However, the precise role of Mn^2+^ in the molecular structure and catalytic process of CLDz2 need to be investigated in detail by high-resolution characterization (e.g. X-ray crystallography and NMR spectroscopy) in the future [31–33]. Sequence truncation and analysis were conducted to confirm the existence of the predicted secondary-structure elements, and identify the possible Watson-Crick base-pairing regions. We also performed reselections from partially randomized DNA pool on the basis of the original CLDz2 sequence to isolate functionally essential nucleotides. Because CLDz2 can be tailored to use variants of an acceptor DNA by altering the sequence of base-pairing region at the 3′ end, this methodology should be employed as an alternative and versatile approach to the chemical synthesis of a series of single-stranded linear DNAs. However, the range of DNA sequences that can be click-ligated is limited by the requirement of the CLDz2 sequence at the 5′ end. Future engineering efforts should evolve more versatile deoxyribozymes that catalyzes the ligation of DNA independent of the DNA sequence. Finally, CLDz2 was used to prepare single-stranded circular DNA with an unnatural backbone that is tolerated during rolling circle amplification. CLDz2-directed chemical ligation offers the advantage in terms of high circularization selectivity. Previously, a ligated DNA strand containing an unnatural T-triazole-T linkage has been synthesised by CuAAC between an oligonucleotide functionalized with 3′-azido-dT and another with 5′-propargylamido dT [34]. The resultant click-ligated DNA strand was used as an efficient template during PCR amplification. This suggests that it could be a candidate as a universal base involved in DNA library synthesis and new click-ligating deoxyribozymes that can be conveniently produced by *in vitro* selection, although further work must be carried out to verify this. Our ongoing efforts seek to understand the more detailed mechanisms of catalysis by high-resolution characterization, and to allow remarkable manipulation of functional nucleotides and reaction rate or yield. We envision that the work described here will broaden the reaction scope and the practical applications of DNA catalysis.

## Supplementary Material

gkaf991_Supplemental_File

## Data Availability

The data underlying this article are available in the article and in its online supplementary material.

## References

[1] Breaker RR, Joyce GF A DNA enzyme that cleaves RNA. Chem Biol. 1994; 1:223–9. 10.1016/1074-5521(94)90014-0. 9383394

[2] McConnell EM, Cozma I, Mou Q et al. Bio-sensing with DNAzymes. Chem Soc Rev. 2021; 50:8954–94. 10.1039/D1CS00240F. 34227631 PMC9136875

[3] Ponce-Salvatierra A, Wawrzyniak-Turek K, Steuerwald U et al. Crystal structure of a DNA catalyst. Nature. 2016; 529:231–4. 10.1038/nature16471. 26735012

[4] Chang D, Zakaria S, Samani SE et al. Functional nucleic acids for pathogenic bacteria detection. Acc Chem Res. 2021; 54:3540–9. 10.1021/acs.accounts.1c00355. 34478272

[5] Zhou Q, Zhang G, Wu Y et al. In vitro selection of M^2+^-independent, fast-responding acidic deoxyribozymes for bacterial detection. J Am Chem Soc. 2023; 145:21370–7. 10.1021/jacs.3c06155. 37683187

[6] Wang Y, Wang Y, Song D et al. An RNA-cleaving threose nucleic acid enzyme capable of single point mutation discrimination. Nat Chem. 2022; 14:350–9. 10.1038/s41557-021-00847-3. 34916596

[7] Boyd R, Kennebeck M, Miranda A et al. Site-specific N-alkylation of DNA oligonucleotide nucleobases by DNAzyme-catalyzed reductive amination. Nucleic Acids Res. 2024; 52:8702–16. 10.1093/nar/gkae639. 39051544 PMC11347174

[8] Sednev MV, Mykhailiuk V, Choudhury P et al. N6-methyladenosine-sensitive RNA-cleaving deoxyribozymes. Angew Chem Int Ed. 2018; 57:15117–21. 10.1002/anie.201808745. 30276938

[9] Zhang C, Li Q, Xu T et al. New DNA-hydrolyzing DNAs isolated from an ssDNA library carrying a terminal hybridization stem. Nucleic Acids Res. 2021; 49:6364–74. 10.1093/nar/gkab439. 34057476 PMC8216280

[10] Chen B, Yu X, Gao T et al. Selection of allosteric DNAzymes that can sense phenylalanine by expression-SELEX. Nucleic Acids Res. 2023; 51:e66–10.1093/nar/gkad424. 37207331 PMC10287898

[11] El-Sagheer AH, Brown T Click nucleic acid ligation: applications in biology and nanotechnology. Acc Chem Res. 2012; 45:1258–67. 10.1021/ar200321n. 22439702 PMC3423825

[12] Fantoni NF, El-Sagheer AH, Brown T A hitchhiker's guide to click-chemistry with nucleic acids. Chem Rev. 2021; 121:7122–54. 10.1021/acs.chemrev.0c00928. 33443411

[13] Li J, Mohammed-Elsabagh M, Paczkowski F et al. Circular nucleic acids: discovery, functions and applications. ChemBioChem. 2020; 21:1547–66. 10.1002/cbic.202000003. 32176816

[14] Valero J, Lohmann F, Famulok M Interlocked DNA topologies for nanotechnology. Curr Opin Biotechnol. 2017; 48:159–67. 10.1016/j.copbio.2017.04.002. 28505598

[15] Flynn-Charlebois A, Wang Y, Prior TK et al. Deoxyribozymes with 2’-5’ RNA ligase activity. J Am Chem Soc. 2003; 125:2444–54. 10.1021/ja028774y. 12603132

[16] Flynn-Charlebois A, Prior TK, Hoadley KA et al. In vitro evolution of an RNA-cleaving DNA enzyme into an RNA ligase switches the selectivity from 3’-5’ to 2’-5’. J Am Chem Soc. 2003; 125:5346–50. 10.1021/ja0340331. 12720447

[17] Wang J, Han F, Zou Y et al. A threose nucleic acid (TNA) enzyme catalyzing native 3’-5’ ligation of RNA. J Am Chem Soc. 2025; 147:18349–58. 10.1021/jacs.5c07235. 40372316

[18] Wang Y, Silverman SK Deoxyribozymes that synthesize branched and lariat RNA. J Am Chem Soc. 2003; 125:6880–1. 10.1021/ja035150z. 12783536

[19] Pratico E, Wang Y, Silverman S A deoxyribozyme that synthesizes 2’, 5’ -branched RNA with any branch-site nucleotide. Nucleic Acids Res. 2005; 33:3503–12. 10.1093/nar/gki656. 15967808 PMC1153712

[20] Wang Y, Wang Y, Song D et al. A threose nucleic acid enzyme with RNA ligase activity. J Am Chem Soc. 2021; 143:8154–63. 10.1021/jacs.1c02895. 34028252

[21] Cuenoud B, Szostak JW A DNA metalloenzyme with DNA ligase activity. Nature. 1995; 375:611–4. 10.1038/375611a0. 7791880

[22] Sreedhara A, Li Y, Breaker RR Ligating DNA with DNA. J Am Chem Soc. 2004; 126:3454–60. 10.1021/ja039713i. 15025472

[23] Wang F, Lu C, Willner I From cascaded catalytic nucleic acids to enzyme−DNA nanostructures: controlling reactivity, sensing, logic operations, and assembly of complex structures. Chem Rev. 2014; 114:2881–941. 10.1021/cr400354z. 24576227

[24] Liu K, Lat P, Yu H et al. CLICK-17, a DNA enzyme that harnesses ultra-low concentrations of either Cu^+^ or Cu^2+^ to catalyze the azide-alkyne ‘click’ reaction in water. Nucleic Acids Res. 2020; 48:7356–70. 32520335 10.1093/nar/gkaa502 PMC7367168

[25] El-Sagheer AH, Brown T Click chemistry with DNA. Chem Soc Rev. 2010; 39:1388–405. 10.1039/b901971p. 20309492

[26] Rostovtsev VV, Green LG, Fokin VV et al. A stepwise huisgen cycloaddition process: copper(I)-catalyzed regioselective ligation of azides and terminal alkynes. Angew Chem Int Ed. 2002; 41:2596–9. 10.1002/1521-3773(20020715)41:14<2596::AID-ANIE2596>3.0.CO;2-4. 12203546

[27] Tornoe CW, Christensen C, Meldal M Peptidotriazoles on solid phase: 1,2,3-triazoles by regiospecific copper(I)-catalyzed 1,3-dipolar cycloadditions of terminal alkynes to azides. J Org Chem. 2002; 67:3057–64. 10.1021/jo011148j. 11975567

[28] Kolb HC, Finn MG, Sharpless KB Click Chemistry: diverse chemical function from a few good reactions. Angew Chem Int Ed. 2001; 40:2004–21. 10.1002/1521-3773(20010601)40:11<2004::AID-ANIE2004>3.0.CO;2-5. 11433435

[29] An R, Li Q, Fan Y et al. Highly efficient preparation of single-stranded DNA rings by T4 ligase at abnormally low Mg(II) concentration. Nucleic Acids Res. 2017; 45:e139 10.1093/nar/gkx553. 28655200 PMC5587803

[30] Cui Y, Han X, An R et al. Terminal hairpin in oligonucleotide dominantly prioritizes intramolecular cyclization by T4 ligase over intermolecular polymerization: an exclusive methodology for producing ssDNA rings. Nucleic Acids Res. 2018; 46:e132. 30169701 10.1093/nar/gky769 PMC6294566

[31] Ponce-Salvatierra A, Wawrzyniak-Turek K, Steuerwald U et al. Crystal structure of a DNA catalyst. Nature. 2016; 529:231–4. 10.1038/nature16471. 26735012

[32] Borggräfe J, Victor J, Rosenbach H et al. Time-resolved structural analysis of an RNA-cleaving DNA catalyst. Nature. 2022; 601:144–9. 10.1038/s41586-021-04225-4. 34949858

[33] Liu H, Yu X, Chen Y et al. Crystal structure of an RNA-cleaving DNAzyme. Nat Commun. 2017; 8:2006 10.1038/s41467-017-02203-x. 29222499 PMC5722873

[34] El-Sagheer AH, Brown T Synthesis and polymerase chain reaction amplification of DNA strands containing an unnatural triazole linkage. J Am Chem Soc. 2009; 131:3958–64. 10.1021/ja8065896. 19292490

