# Supplementary material for: Click DNA ligation with deoxyribozyme
Source: Nucleic Acids Res. 2025 Oct 2;53(18):gkaf991. doi: 10.1093/nar/gkaf991 (PMC12489470; doi:10.1093/nar/gkaf991)
Supplement: gkaf991_Supplemental_File [file gkaf991_supplemental_file.docx]

**Supplementary Table 1.** DNA sequences used in this work

| **DNA oligonucleotide** | **Sequence (5’-3’)** |
| --- | --- |
| DP1 (DNA pool, 82 nt) | Hexynyl-CAGGT CCATC GAGTG GTAGG A-N_40_-TCGCA CTGCT CCTGA ACGTA C |
| Forward primer FP1 (21 nt) | Hexynyl-CAGGT CCATC GAGTG GTAGG A |
| Reverse primer RP1 (21 nt) | GTACG TTCAG GAGCA GTGCG A |
| Reverse primer RP2 (42 nt) | A21/Spacer C3/GTACG TTCAG GAGCA GTGCG A |
| AD1 (acceptor DNA ,14 nt) | FAM-CAGGA GCAGT GCGA-N_3_ |
| CLSS1 (82 nt) | Hexynyl-CAGGT CCATC GAGTG GTAGG AATCG GGCGA GACGTA GATCG ATGGG AGATAC AGTAG ATCCT CGCAC TGCTC CTGAA CGTAC |
| LDNA1 (96 nt) | Hexynyl-CAGGT CCATC GAGTG G/i6FAMdT/AGG ACACT AACCA TTACG GGGCG AATAA TCGCT ACTCT AGTCC TTCGC ACTGC TCCTG AACGA TCCAG GAGCA GTGCG A-N_3_ |
| RCA primer (15 nt) | AGGAC TAGAG TAGCG |

**Supplementary Table 2.** High-throughput sequencing results from round 11­th pool

| **Name** | **Seq of N_40_ (5’-3’)** | **Ratio** |
| --- | --- | --- |
| CLDz1 | CAAGG GCCGA GTGCG TCGGG TCTCG TAAGA GGGTA CACCT | 22.76% |
| CLDz2 | CACTA ACCAT TACGG GGCGA ATAAT CGCTA CTCTA  GTCCT | 20.07% |
| CLDz3 | GCAGA CACTG CAAGA AGGAC ATGCG TGTCG CCCGG TGGGG | 16.52% |
| CLDz4 | ATTGC CGTAC CACGT GCAGC GAGGT CGTCA TGGGT TGGTG | 7.40% |
| CLDz5 | GCGGT CTATG CGAGT CCTCA CATAC GGTTC CAGGA TCGAT | 6.48% |
| CLDz6 | GCAGA CACTG CAGGA AGGAC ATGCG TGTCG CCCGG TGGGG | 1.09% |
| CLDz7 | GGAGG AGGCG CAGAT GTTTT TCAAA TGTCG GTCGG TGGGC | 1.09% |
| CLDz8 | GCAGA CACTG CAAGA AGGAC GTGCG TGTCG CCCGG TGGGG | 1.06% |
| CLDz9 | GCAGA CACAG CAAGA AGGAC ATGCG TGTCG CCCGG TGGGG | 0.99% |
| CLDz10 | GCACT GCGGA TGGGC TAGCG CAAGT TATGT GATGA TCACC | 0.8% |

**Note:** Each sequence also contains Hexynyl-CAGGT CCATC GAGTG GTAGG A at the 5' end and TCGCA CTGCT CCTGA ACGTA C at the 3' end.

**Supplementary Table 3.** Comparison of CLDz2 with previously reported DNA-ligating deoxyribozymes.

| **DNAzyme name** | ***k_obs_*** | **metal concentration** | **pH** |
| --- | --- | --- | --- |
| E47 | 3.4 h^-1^ | Zn^2+^ (4 mM)/Cu^2+^ (10 μM) | 7.2 |
| L115 | 6.0 × 10^-3^ h^-1^ | Mg^2+^ (15 mM), Ca^2+^ (5 mM) and Mn^2+^ (10 mM) | 7.0 |
| CLDz2 | 2.4 × 10^-2^ h^-1^ | Mn^2+^ (10 mM) | 7.0 |


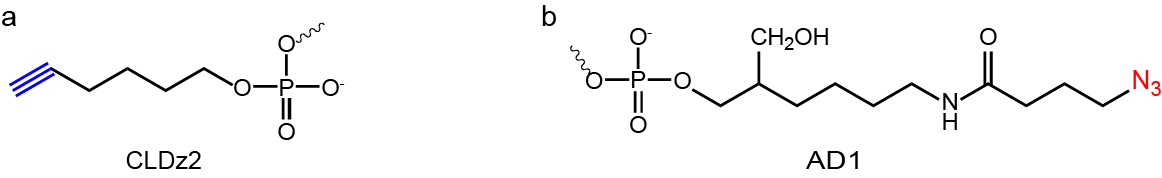


[**Supplementary**](https://oup.silverchair-cdn.com/oup/backfile/Content_public/Journal/nar/53/11/10.1093_nar_gkaf485/1/gkaf485_supplemental_files.zip?Expires=1753205889&Signature=rVSjWwEmbwT0fSZOBEnmvh76IRhXdvs~uC46NZ0r-DhTdgJRt1pgRAlKm~8cTkOfNNUVhGLyVFuh58Ob3v0~et0ayTCgZjPJSTtxr3XfEcULbQsuw2ZR1OWNRLNfvtyu2QJg21MNvrJtMOEw8OftiI8Ri96ZR1DY0~zUdgkDQ~JoHwG4W6uPh83KBT2WgZCg6tlmDwMtAdmM~QEGRVQCMxCDe0SrOS9umcTOwelAVEpKa~bXCoSKZOnzhi2~FjYCuS7pEnMLP6I1dMVoUa~ah2-cHd4KC9Y56D-xcTAH40GBllbH2jKXMGSfeka57rhH~f9tKumiue1dk-IRgtpGig__&Key-Pair-Id=APKAIE5G5CRDK6RD3PGA) **Figure 1.** Chemical structures of (a) 5’ alkyne group and (b) 3’ azide group.

**
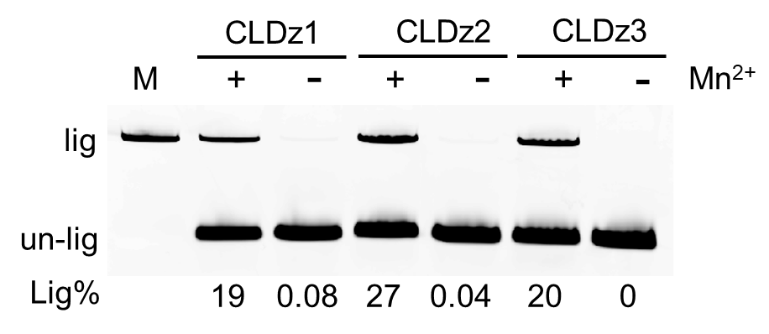
**

**[Supplementary](https://oup.silverchair-cdn.com/oup/backfile/Content_public/Journal/nar/53/11/10.1093_nar_gkaf485/1/gkaf485_supplemental_files.zip?Expires=1753205889&Signature=rVSjWwEmbwT0fSZOBEnmvh76IRhXdvs~uC46NZ0r-DhTdgJRt1pgRAlKm~8cTkOfNNUVhGLyVFuh58Ob3v0~et0ayTCgZjPJSTtxr3XfEcULbQsuw2ZR1OWNRLNfvtyu2QJg21MNvrJtMOEw8OftiI8Ri96ZR1DY0~zUdgkDQ~JoHwG4W6uPh83KBT2WgZCg6tlmDwMtAdmM~QEGRVQCMxCDe0SrOS9umcTOwelAVEpKa~bXCoSKZOnzhi2~FjYCuS7pEnMLP6I1dMVoUa~ah2-cHd4KC9Y56D-xcTAH40GBllbH2jKXMGSfeka57rhH~f9tKumiue1dk-IRgtpGig__&Key-Pair-Id=APKAIE5G5CRDK6RD3PGA) Figure 2.** 10% dPAGE analysis of the click-ligating activity of the top 3 sequences under in vitro selection conditions with and without Mn^2+^. Reaction time: 24 h, lig: ligated product, un-lig: un-ligated product, Lig%: ligation percentage, M: marker.

**Experimental details:** The top 3 sequences (named CLDz1, CLDz2 and CLDz3, listed in Supplementary Table 2) were chemically synthesized. Their click-ligating activity to AD1 was tested as follows: 6 µL of each sequence (10 μM), 2 µL of AD1 (10 μM) and 50 µL of 2× SB-a were mixed and diluted to 90 µL using ddH_2_O. After incubation at 90°C for 5 min, the mixture was cooled at RT for 10 min. 10 μL of 200 mM MnCl_2_ (or DNase/RNase-free water as a control) was added into the above mixture and incubated at 30°C for 24 h. Followed by ethanol precipitation, the resultant products were analyzed by 10% dPAGE.


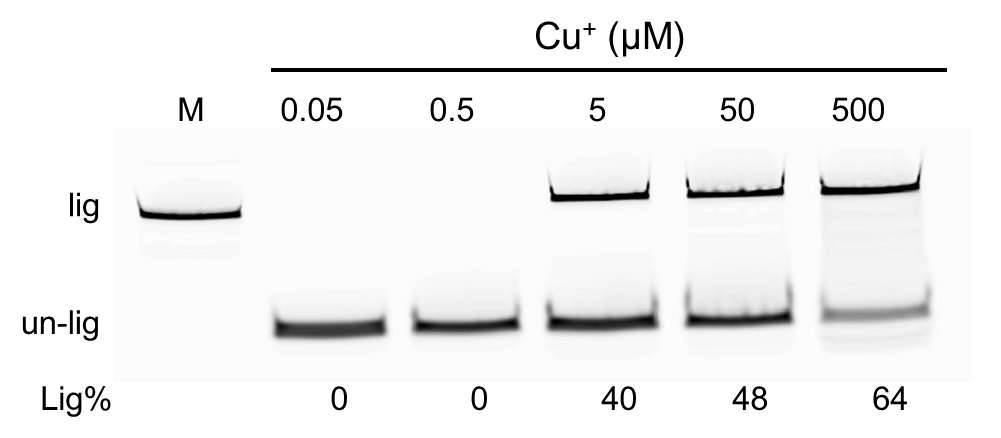


[**Supplementary**](https://oup.silverchair-cdn.com/oup/backfile/Content_public/Journal/nar/53/11/10.1093_nar_gkaf485/1/gkaf485_supplemental_files.zip?Expires=1753205889&Signature=rVSjWwEmbwT0fSZOBEnmvh76IRhXdvs~uC46NZ0r-DhTdgJRt1pgRAlKm~8cTkOfNNUVhGLyVFuh58Ob3v0~et0ayTCgZjPJSTtxr3XfEcULbQsuw2ZR1OWNRLNfvtyu2QJg21MNvrJtMOEw8OftiI8Ri96ZR1DY0~zUdgkDQ~JoHwG4W6uPh83KBT2WgZCg6tlmDwMtAdmM~QEGRVQCMxCDe0SrOS9umcTOwelAVEpKa~bXCoSKZOnzhi2~FjYCuS7pEnMLP6I1dMVoUa~ah2-cHd4KC9Y56D-xcTAH40GBllbH2jKXMGSfeka57rhH~f9tKumiue1dk-IRgtpGig__&Key-Pair-Id=APKAIE5G5CRDK6RD3PGA) **Figure 3.** 10% dPAGE analysis of the click-ligating activity of CLDz2 in the presence of different concentrations of Cu^+^. Reaction time: 24 h, lig: ligated product, un-lig: un-ligated product, Lig%: ligation percentage, M: marker.

**Experimental details:** CuSO_4_ and THPTA were pre-mixed at a molar ratio of 1:2. The final concentration of CuSO_4_ was set as 0.05 μM, 0.5 μM, 5 μM, 50 μM and 500 μM, respectively. 10 μL of the mixture, 10 μL of ascorbic acid (20 μM), 6 μL of CLDz2 (10 μM), 2 μL of AD1 (10 μM), and 50 μL of 2× SB-b were mixed and diluted to 100 μL using ddH_2_O. After incubation at 30°C for 24 h, the reaction was stopped by ethanol precipitation. Finally, the obtained products were analyzed using 10% dPAGE.

**
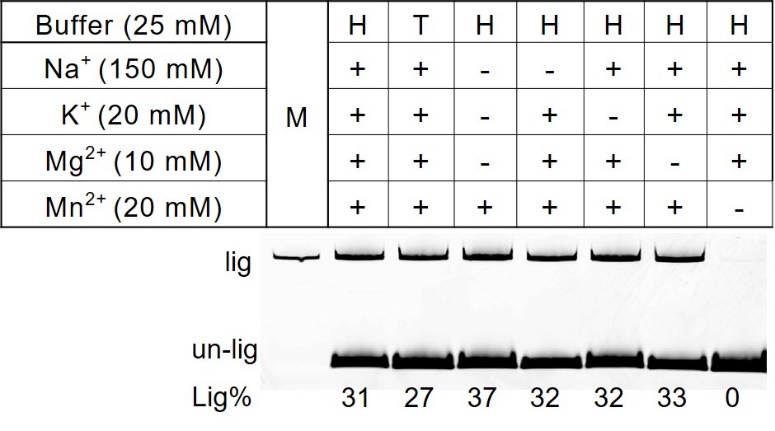
**

[**Supplementary**](https://oup.silverchair-cdn.com/oup/backfile/Content_public/Journal/nar/53/11/10.1093_nar_gkaf485/1/gkaf485_supplemental_files.zip?Expires=1753205889&Signature=rVSjWwEmbwT0fSZOBEnmvh76IRhXdvs~uC46NZ0r-DhTdgJRt1pgRAlKm~8cTkOfNNUVhGLyVFuh58Ob3v0~et0ayTCgZjPJSTtxr3XfEcULbQsuw2ZR1OWNRLNfvtyu2QJg21MNvrJtMOEw8OftiI8Ri96ZR1DY0~zUdgkDQ~JoHwG4W6uPh83KBT2WgZCg6tlmDwMtAdmM~QEGRVQCMxCDe0SrOS9umcTOwelAVEpKa~bXCoSKZOnzhi2~FjYCuS7pEnMLP6I1dMVoUa~ah2-cHd4KC9Y56D-xcTAH40GBllbH2jKXMGSfeka57rhH~f9tKumiue1dk-IRgtpGig__&Key-Pair-Id=APKAIE5G5CRDK6RD3PGA) **Figure 4.** 10% dPAGE analysis of the click-ligating activity of CLDz2 under different buffer and metal conditions. Reaction time: 24 h, lig: ligated product, un-lig: un-ligated product, Lig%: ligation percentage, M: marker, T: Tris-HCl, H: HEPES.

**Experimental details:** The protocol for the metal dependency analysis of CLDz2 is similar to the one described in [Supplementary](https://oup.silverchair-cdn.com/oup/backfile/Content_public/Journal/nar/53/11/10.1093_nar_gkaf485/1/gkaf485_supplemental_files.zip?Expires=1753205889&Signature=rVSjWwEmbwT0fSZOBEnmvh76IRhXdvs~uC46NZ0r-DhTdgJRt1pgRAlKm~8cTkOfNNUVhGLyVFuh58Ob3v0~et0ayTCgZjPJSTtxr3XfEcULbQsuw2ZR1OWNRLNfvtyu2QJg21MNvrJtMOEw8OftiI8Ri96ZR1DY0~zUdgkDQ~JoHwG4W6uPh83KBT2WgZCg6tlmDwMtAdmM~QEGRVQCMxCDe0SrOS9umcTOwelAVEpKa~bXCoSKZOnzhi2~FjYCuS7pEnMLP6I1dMVoUa~ah2-cHd4KC9Y56D-xcTAH40GBllbH2jKXMGSfeka57rhH~f9tKumiue1dk-IRgtpGig__&Key-Pair-Id=APKAIE5G5CRDK6RD3PGA) Figure 1, except for: (1) SB-a was replaced by SB-b; (2) Mn^2+^ was replaced by different metal ions. The protocol for the buffer dependency analysis of CLDz2 is similar to the one described in [Supplementary](https://oup.silverchair-cdn.com/oup/backfile/Content_public/Journal/nar/53/11/10.1093_nar_gkaf485/1/gkaf485_supplemental_files.zip?Expires=1753205889&Signature=rVSjWwEmbwT0fSZOBEnmvh76IRhXdvs~uC46NZ0r-DhTdgJRt1pgRAlKm~8cTkOfNNUVhGLyVFuh58Ob3v0~et0ayTCgZjPJSTtxr3XfEcULbQsuw2ZR1OWNRLNfvtyu2QJg21MNvrJtMOEw8OftiI8Ri96ZR1DY0~zUdgkDQ~JoHwG4W6uPh83KBT2WgZCg6tlmDwMtAdmM~QEGRVQCMxCDe0SrOS9umcTOwelAVEpKa~bXCoSKZOnzhi2~FjYCuS7pEnMLP6I1dMVoUa~ah2-cHd4KC9Y56D-xcTAH40GBllbH2jKXMGSfeka57rhH~f9tKumiue1dk-IRgtpGig__&Key-Pair-Id=APKAIE5G5CRDK6RD3PGA) Figure 1, except for: HEPES was replaced by Tris-HCl.


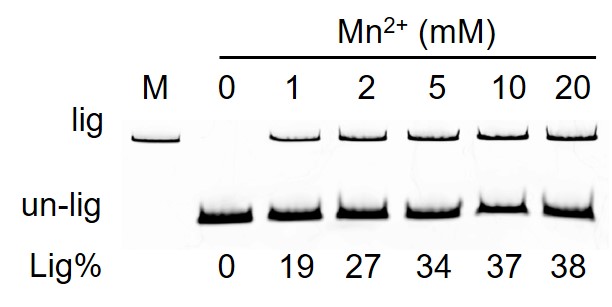


[**Supplementary**](https://oup.silverchair-cdn.com/oup/backfile/Content_public/Journal/nar/53/11/10.1093_nar_gkaf485/1/gkaf485_supplemental_files.zip?Expires=1753205889&Signature=rVSjWwEmbwT0fSZOBEnmvh76IRhXdvs~uC46NZ0r-DhTdgJRt1pgRAlKm~8cTkOfNNUVhGLyVFuh58Ob3v0~et0ayTCgZjPJSTtxr3XfEcULbQsuw2ZR1OWNRLNfvtyu2QJg21MNvrJtMOEw8OftiI8Ri96ZR1DY0~zUdgkDQ~JoHwG4W6uPh83KBT2WgZCg6tlmDwMtAdmM~QEGRVQCMxCDe0SrOS9umcTOwelAVEpKa~bXCoSKZOnzhi2~FjYCuS7pEnMLP6I1dMVoUa~ah2-cHd4KC9Y56D-xcTAH40GBllbH2jKXMGSfeka57rhH~f9tKumiue1dk-IRgtpGig__&Key-Pair-Id=APKAIE5G5CRDK6RD3PGA) **Figure 5.** 10% dPAGE analysis of the click-ligating activity of CLDz2 in the presence of different concentrations of Mn^2+^. Reaction time: 24 h, lig: ligated product, un-lig: un-ligated product, Lig%: ligation percentage, M: marker.

**Experimental details:** The protocol for the Mn^2+^ concentration dependency analysis of CLDz2 is similar to the one described in [Supplementary](https://oup.silverchair-cdn.com/oup/backfile/Content_public/Journal/nar/53/11/10.1093_nar_gkaf485/1/gkaf485_supplemental_files.zip?Expires=1753205889&Signature=rVSjWwEmbwT0fSZOBEnmvh76IRhXdvs~uC46NZ0r-DhTdgJRt1pgRAlKm~8cTkOfNNUVhGLyVFuh58Ob3v0~et0ayTCgZjPJSTtxr3XfEcULbQsuw2ZR1OWNRLNfvtyu2QJg21MNvrJtMOEw8OftiI8Ri96ZR1DY0~zUdgkDQ~JoHwG4W6uPh83KBT2WgZCg6tlmDwMtAdmM~QEGRVQCMxCDe0SrOS9umcTOwelAVEpKa~bXCoSKZOnzhi2~FjYCuS7pEnMLP6I1dMVoUa~ah2-cHd4KC9Y56D-xcTAH40GBllbH2jKXMGSfeka57rhH~f9tKumiue1dk-IRgtpGig__&Key-Pair-Id=APKAIE5G5CRDK6RD3PGA) Figure 1, except for: (1) SB-a was replaced by SB-b; (2) the final Mn^2+^ concentration was set as 0, 1 mM, 5 mM, 10 mM and 20 mM, respectively.


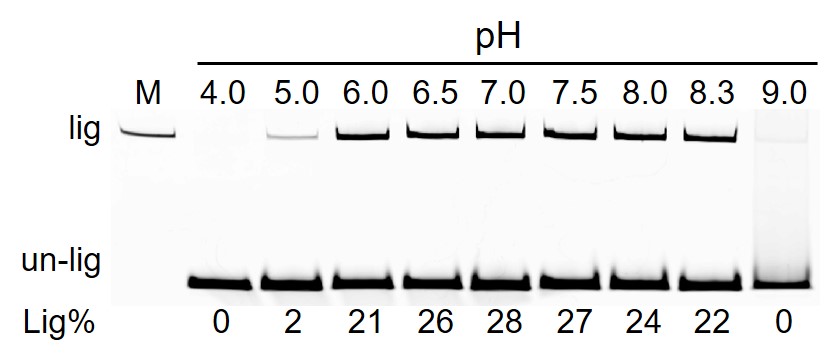


[**Supplementary**](https://oup.silverchair-cdn.com/oup/backfile/Content_public/Journal/nar/53/11/10.1093_nar_gkaf485/1/gkaf485_supplemental_files.zip?Expires=1753205889&Signature=rVSjWwEmbwT0fSZOBEnmvh76IRhXdvs~uC46NZ0r-DhTdgJRt1pgRAlKm~8cTkOfNNUVhGLyVFuh58Ob3v0~et0ayTCgZjPJSTtxr3XfEcULbQsuw2ZR1OWNRLNfvtyu2QJg21MNvrJtMOEw8OftiI8Ri96ZR1DY0~zUdgkDQ~JoHwG4W6uPh83KBT2WgZCg6tlmDwMtAdmM~QEGRVQCMxCDe0SrOS9umcTOwelAVEpKa~bXCoSKZOnzhi2~FjYCuS7pEnMLP6I1dMVoUa~ah2-cHd4KC9Y56D-xcTAH40GBllbH2jKXMGSfeka57rhH~f9tKumiue1dk-IRgtpGig__&Key-Pair-Id=APKAIE5G5CRDK6RD3PGA) **Figure 6.** 10% dPAGE analysis of the click-ligating activity of CLDz2 at different pH values under in vitro selection conditions. Reaction time: 24 h. lig: ligated product, un-lig: un-ligated product, Lig%: ligation percentage, M: marker.

**Experimental details:** The protocol for pH dependency analysis of CLDz2 is similar to the one described in [Supplementary](https://oup.silverchair-cdn.com/oup/backfile/Content_public/Journal/nar/53/11/10.1093_nar_gkaf485/1/gkaf485_supplemental_files.zip?Expires=1753205889&Signature=rVSjWwEmbwT0fSZOBEnmvh76IRhXdvs~uC46NZ0r-DhTdgJRt1pgRAlKm~8cTkOfNNUVhGLyVFuh58Ob3v0~et0ayTCgZjPJSTtxr3XfEcULbQsuw2ZR1OWNRLNfvtyu2QJg21MNvrJtMOEw8OftiI8Ri96ZR1DY0~zUdgkDQ~JoHwG4W6uPh83KBT2WgZCg6tlmDwMtAdmM~QEGRVQCMxCDe0SrOS9umcTOwelAVEpKa~bXCoSKZOnzhi2~FjYCuS7pEnMLP6I1dMVoUa~ah2-cHd4KC9Y56D-xcTAH40GBllbH2jKXMGSfeka57rhH~f9tKumiue1dk-IRgtpGig__&Key-Pair-Id=APKAIE5G5CRDK6RD3PGA) Figure 1, except for: 50 mM HEPES in 2× SB-a was replaced by 50 mM other buffering reagents. NaAC for pH 4.0 and 5.0; MES for 6.0; HEPES for 6.5, 7.0 and 7.5; Tris-HCl for pH 8.0, 8.3 and 9.0.

**
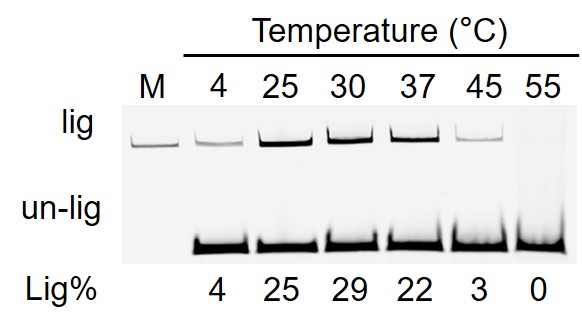
**

[**Supplementary**](https://oup.silverchair-cdn.com/oup/backfile/Content_public/Journal/nar/53/11/10.1093_nar_gkaf485/1/gkaf485_supplemental_files.zip?Expires=1753205889&Signature=rVSjWwEmbwT0fSZOBEnmvh76IRhXdvs~uC46NZ0r-DhTdgJRt1pgRAlKm~8cTkOfNNUVhGLyVFuh58Ob3v0~et0ayTCgZjPJSTtxr3XfEcULbQsuw2ZR1OWNRLNfvtyu2QJg21MNvrJtMOEw8OftiI8Ri96ZR1DY0~zUdgkDQ~JoHwG4W6uPh83KBT2WgZCg6tlmDwMtAdmM~QEGRVQCMxCDe0SrOS9umcTOwelAVEpKa~bXCoSKZOnzhi2~FjYCuS7pEnMLP6I1dMVoUa~ah2-cHd4KC9Y56D-xcTAH40GBllbH2jKXMGSfeka57rhH~f9tKumiue1dk-IRgtpGig__&Key-Pair-Id=APKAIE5G5CRDK6RD3PGA) **Figure 7.** 10% dPAGE analysis of the click-ligating activity of CLDz2 at different temperatures under in vitro selection conditions. Reaction time: 24 h, lig: ligated product, un-lig: un-ligated product, Lig%: ligation percentage, M: marker.

**Experimental details:** The protocol for temperature dependency analysis of CLDz2 is similar to the one described in Supplementary Figure 1, except for: reaction temperature was set as 4°C, 25°C, 30°C, 37°C, 45°C and 55°C, respectively.


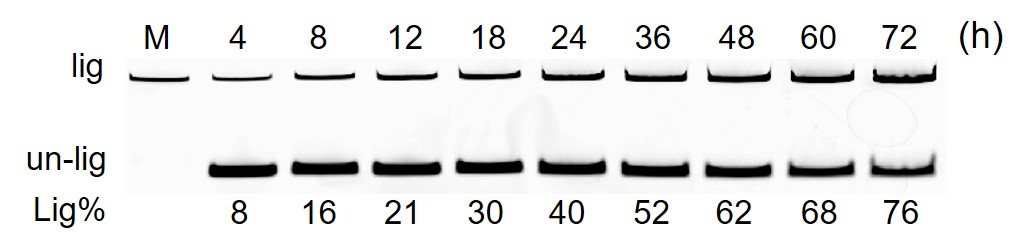


[**Supplementary**](https://oup.silverchair-cdn.com/oup/backfile/Content_public/Journal/nar/53/11/10.1093_nar_gkaf485/1/gkaf485_supplemental_files.zip?Expires=1753205889&Signature=rVSjWwEmbwT0fSZOBEnmvh76IRhXdvs~uC46NZ0r-DhTdgJRt1pgRAlKm~8cTkOfNNUVhGLyVFuh58Ob3v0~et0ayTCgZjPJSTtxr3XfEcULbQsuw2ZR1OWNRLNfvtyu2QJg21MNvrJtMOEw8OftiI8Ri96ZR1DY0~zUdgkDQ~JoHwG4W6uPh83KBT2WgZCg6tlmDwMtAdmM~QEGRVQCMxCDe0SrOS9umcTOwelAVEpKa~bXCoSKZOnzhi2~FjYCuS7pEnMLP6I1dMVoUa~ah2-cHd4KC9Y56D-xcTAH40GBllbH2jKXMGSfeka57rhH~f9tKumiue1dk-IRgtpGig__&Key-Pair-Id=APKAIE5G5CRDK6RD3PGA) **Figure 8.** Time-dependent click-ligating activity of CLDz2. lig: ligated product, un-lig: un-ligated product, Lig%: ligation percentage, M: marker.


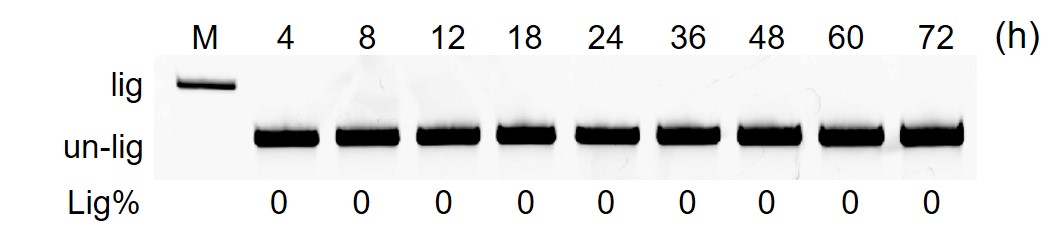


[**Supplementary**](https://oup.silverchair-cdn.com/oup/backfile/Content_public/Journal/nar/53/11/10.1093_nar_gkaf485/1/gkaf485_supplemental_files.zip?Expires=1753205889&Signature=rVSjWwEmbwT0fSZOBEnmvh76IRhXdvs~uC46NZ0r-DhTdgJRt1pgRAlKm~8cTkOfNNUVhGLyVFuh58Ob3v0~et0ayTCgZjPJSTtxr3XfEcULbQsuw2ZR1OWNRLNfvtyu2QJg21MNvrJtMOEw8OftiI8Ri96ZR1DY0~zUdgkDQ~JoHwG4W6uPh83KBT2WgZCg6tlmDwMtAdmM~QEGRVQCMxCDe0SrOS9umcTOwelAVEpKa~bXCoSKZOnzhi2~FjYCuS7pEnMLP6I1dMVoUa~ah2-cHd4KC9Y56D-xcTAH40GBllbH2jKXMGSfeka57rhH~f9tKumiue1dk-IRgtpGig__&Key-Pair-Id=APKAIE5G5CRDK6RD3PGA) **Figure 9.** Time-dependent click-ligating activity of CLSS1. lig: ligated product, un-lig: un-ligated product, Lig%: ligation percentage, M: marker.


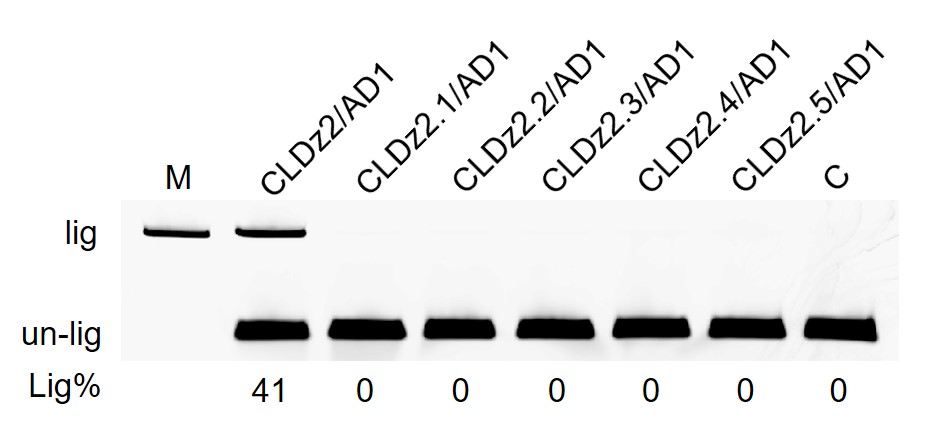


[**Supplementary**](https://oup.silverchair-cdn.com/oup/backfile/Content_public/Journal/nar/53/11/10.1093_nar_gkaf485/1/gkaf485_supplemental_files.zip?Expires=1753205889&Signature=rVSjWwEmbwT0fSZOBEnmvh76IRhXdvs~uC46NZ0r-DhTdgJRt1pgRAlKm~8cTkOfNNUVhGLyVFuh58Ob3v0~et0ayTCgZjPJSTtxr3XfEcULbQsuw2ZR1OWNRLNfvtyu2QJg21MNvrJtMOEw8OftiI8Ri96ZR1DY0~zUdgkDQ~JoHwG4W6uPh83KBT2WgZCg6tlmDwMtAdmM~QEGRVQCMxCDe0SrOS9umcTOwelAVEpKa~bXCoSKZOnzhi2~FjYCuS7pEnMLP6I1dMVoUa~ah2-cHd4KC9Y56D-xcTAH40GBllbH2jKXMGSfeka57rhH~f9tKumiue1dk-IRgtpGig__&Key-Pair-Id=APKAIE5G5CRDK6RD3PGA) **Figure 10.** 10% dPAGE analysis of the click-ligating activity of various CLDz2 variants. Reaction time: 24 h, lig: ligated product, un-lig: un-ligated product, Lig%: ligation percentage, M: marker, C: CLSS1.

.


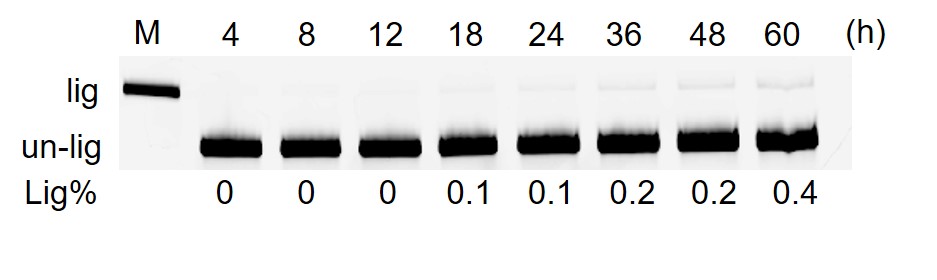


[**Supplementary**](https://oup.silverchair-cdn.com/oup/backfile/Content_public/Journal/nar/53/11/10.1093_nar_gkaf485/1/gkaf485_supplemental_files.zip?Expires=1753205889&Signature=rVSjWwEmbwT0fSZOBEnmvh76IRhXdvs~uC46NZ0r-DhTdgJRt1pgRAlKm~8cTkOfNNUVhGLyVFuh58Ob3v0~et0ayTCgZjPJSTtxr3XfEcULbQsuw2ZR1OWNRLNfvtyu2QJg21MNvrJtMOEw8OftiI8Ri96ZR1DY0~zUdgkDQ~JoHwG4W6uPh83KBT2WgZCg6tlmDwMtAdmM~QEGRVQCMxCDe0SrOS9umcTOwelAVEpKa~bXCoSKZOnzhi2~FjYCuS7pEnMLP6I1dMVoUa~ah2-cHd4KC9Y56D-xcTAH40GBllbH2jKXMGSfeka57rhH~f9tKumiue1dk-IRgtpGig__&Key-Pair-Id=APKAIE5G5CRDK6RD3PGA) **Figure 11.** Time-dependent click-ligating activity of 5’ azide-modified CLDz2.6 with 3’ alkyne-labeled AD1.1. lig: ligated product, un-lig: un-ligated product, Lig%: ligation percentage, M: marker.


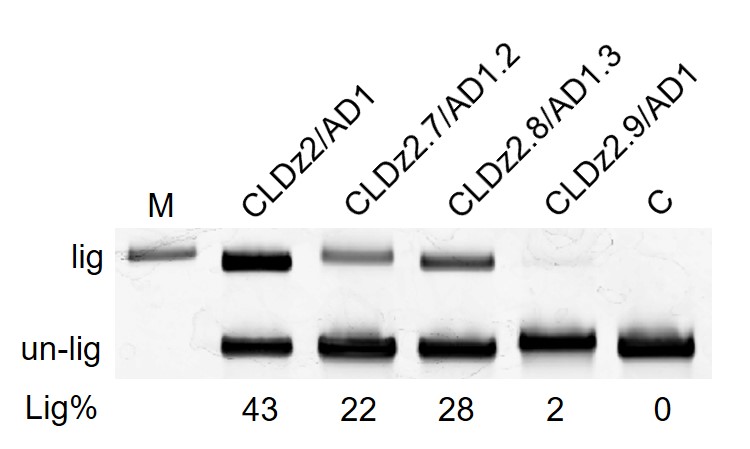


[**Supplementary**](https://oup.silverchair-cdn.com/oup/backfile/Content_public/Journal/nar/53/11/10.1093_nar_gkaf485/1/gkaf485_supplemental_files.zip?Expires=1753205889&Signature=rVSjWwEmbwT0fSZOBEnmvh76IRhXdvs~uC46NZ0r-DhTdgJRt1pgRAlKm~8cTkOfNNUVhGLyVFuh58Ob3v0~et0ayTCgZjPJSTtxr3XfEcULbQsuw2ZR1OWNRLNfvtyu2QJg21MNvrJtMOEw8OftiI8Ri96ZR1DY0~zUdgkDQ~JoHwG4W6uPh83KBT2WgZCg6tlmDwMtAdmM~QEGRVQCMxCDe0SrOS9umcTOwelAVEpKa~bXCoSKZOnzhi2~FjYCuS7pEnMLP6I1dMVoUa~ah2-cHd4KC9Y56D-xcTAH40GBllbH2jKXMGSfeka57rhH~f9tKumiue1dk-IRgtpGig__&Key-Pair-Id=APKAIE5G5CRDK6RD3PGA) **Figure 12.** 10% dPAGE analysis of the click-ligating activity of different CLDz2/AD1 variants at the ligation site. Reaction time: 24 h, lig: ligated product, un-lig: un-ligated product, Lig%: ligation percentage, M: marker, C: CLSS1.


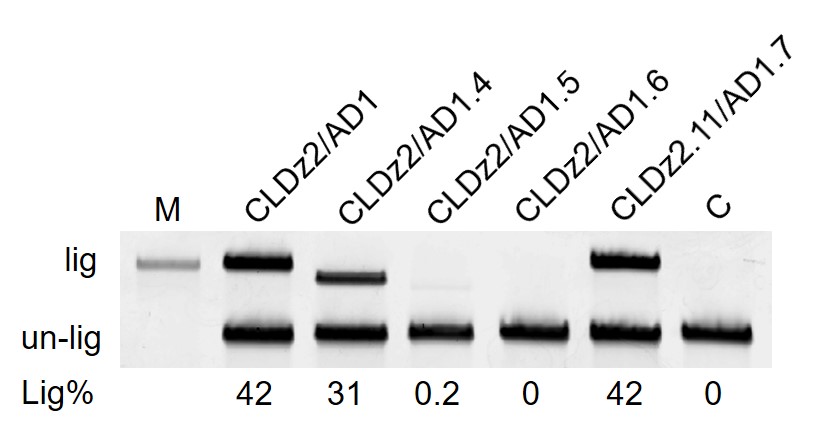


[**Supplementary**](https://oup.silverchair-cdn.com/oup/backfile/Content_public/Journal/nar/53/11/10.1093_nar_gkaf485/1/gkaf485_supplemental_files.zip?Expires=1753205889&Signature=rVSjWwEmbwT0fSZOBEnmvh76IRhXdvs~uC46NZ0r-DhTdgJRt1pgRAlKm~8cTkOfNNUVhGLyVFuh58Ob3v0~et0ayTCgZjPJSTtxr3XfEcULbQsuw2ZR1OWNRLNfvtyu2QJg21MNvrJtMOEw8OftiI8Ri96ZR1DY0~zUdgkDQ~JoHwG4W6uPh83KBT2WgZCg6tlmDwMtAdmM~QEGRVQCMxCDe0SrOS9umcTOwelAVEpKa~bXCoSKZOnzhi2~FjYCuS7pEnMLP6I1dMVoUa~ah2-cHd4KC9Y56D-xcTAH40GBllbH2jKXMGSfeka57rhH~f9tKumiue1dk-IRgtpGig__&Key-Pair-Id=APKAIE5G5CRDK6RD3PGA) **Figure 13.** 10% dPAGE analysis of the click-ligating activity of CLDz2 with various AD1 variants (AD1.4, AD1.5 and AD1.6), and CLDz2.11 with AD1.7. Reaction time: 24 h, lig: ligated product, un-lig: un-ligated product, Lig%: ligation percentage, M: marker, C: CLSS1.


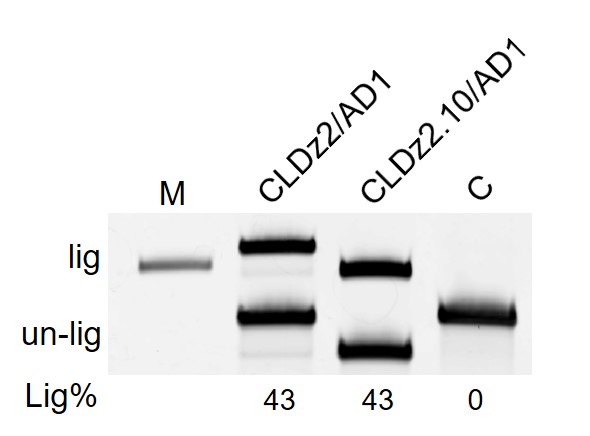


[**Supplementary**](https://oup.silverchair-cdn.com/oup/backfile/Content_public/Journal/nar/53/11/10.1093_nar_gkaf485/1/gkaf485_supplemental_files.zip?Expires=1753205889&Signature=rVSjWwEmbwT0fSZOBEnmvh76IRhXdvs~uC46NZ0r-DhTdgJRt1pgRAlKm~8cTkOfNNUVhGLyVFuh58Ob3v0~et0ayTCgZjPJSTtxr3XfEcULbQsuw2ZR1OWNRLNfvtyu2QJg21MNvrJtMOEw8OftiI8Ri96ZR1DY0~zUdgkDQ~JoHwG4W6uPh83KBT2WgZCg6tlmDwMtAdmM~QEGRVQCMxCDe0SrOS9umcTOwelAVEpKa~bXCoSKZOnzhi2~FjYCuS7pEnMLP6I1dMVoUa~ah2-cHd4KC9Y56D-xcTAH40GBllbH2jKXMGSfeka57rhH~f9tKumiue1dk-IRgtpGig__&Key-Pair-Id=APKAIE5G5CRDK6RD3PGA) **Figure 14.** 10% dPAGE analysis of the click-ligating activity of CDLz2.10 with AD1. Reaction time: 24 h, lig: ligated product, un-lig: un-ligated product, Lig%: ligation percentage, M: marker, C: CLSS1.

[**Supplementary**](https://oup.silverchair-cdn.com/oup/backfile/Content_public/Journal/nar/53/11/10.1093_nar_gkaf485/1/gkaf485_supplemental_files.zip?Expires=1753205889&Signature=rVSjWwEmbwT0fSZOBEnmvh76IRhXdvs~uC46NZ0r-DhTdgJRt1pgRAlKm~8cTkOfNNUVhGLyVFuh58Ob3v0~et0ayTCgZjPJSTtxr3XfEcULbQsuw2ZR1OWNRLNfvtyu2QJg21MNvrJtMOEw8OftiI8Ri96ZR1DY0~zUdgkDQ~JoHwG4W6uPh83KBT2WgZCg6tlmDwMtAdmM~QEGRVQCMxCDe0SrOS9umcTOwelAVEpKa~bXCoSKZOnzhi2~FjYCuS7pEnMLP6I1dMVoUa~ah2-cHd4KC9Y56D-xcTAH40GBllbH2jKXMGSfeka57rhH~f9tKumiue1dk-IRgtpGig__&Key-Pair-Id=APKAIE5G5CRDK6RD3PGA) **Figure 15.** Re-selection progress. The reaction time and ligation percentage (Lig%) for each round are indicated.

**
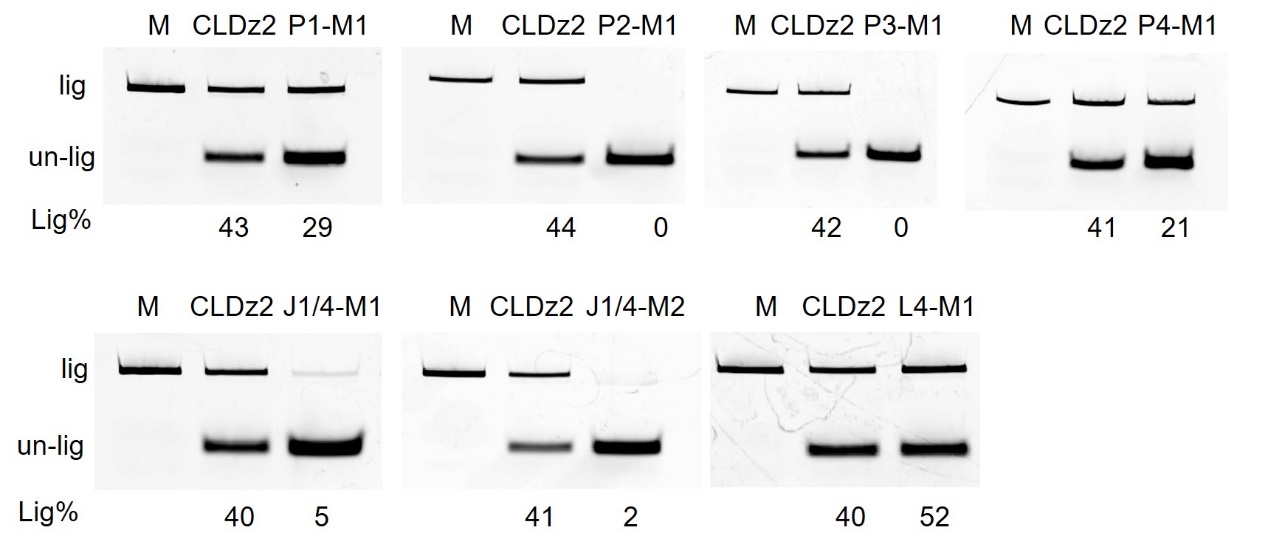
**

[**Supplementary**](https://oup.silverchair-cdn.com/oup/backfile/Content_public/Journal/nar/53/11/10.1093_nar_gkaf485/1/gkaf485_supplemental_files.zip?Expires=1753205889&Signature=rVSjWwEmbwT0fSZOBEnmvh76IRhXdvs~uC46NZ0r-DhTdgJRt1pgRAlKm~8cTkOfNNUVhGLyVFuh58Ob3v0~et0ayTCgZjPJSTtxr3XfEcULbQsuw2ZR1OWNRLNfvtyu2QJg21MNvrJtMOEw8OftiI8Ri96ZR1DY0~zUdgkDQ~JoHwG4W6uPh83KBT2WgZCg6tlmDwMtAdmM~QEGRVQCMxCDe0SrOS9umcTOwelAVEpKa~bXCoSKZOnzhi2~FjYCuS7pEnMLP6I1dMVoUa~ah2-cHd4KC9Y56D-xcTAH40GBllbH2jKXMGSfeka57rhH~f9tKumiue1dk-IRgtpGig__&Key-Pair-Id=APKAIE5G5CRDK6RD3PGA) **Figure 16.** 10% dPAGE analysis of the click-ligating activity of various CDLz2 mutants. Reaction time: 24 h, lig: ligated product, un-lig: un-ligated product, Lig%: ligation percentage, M: marker.


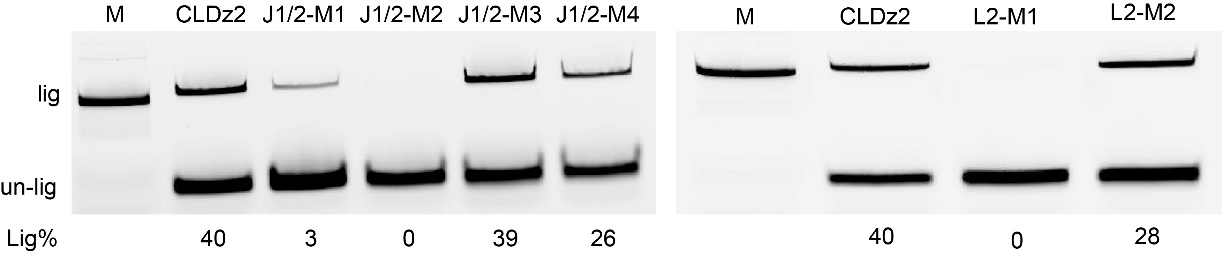


[**Supplementary**](https://oup.silverchair-cdn.com/oup/backfile/Content_public/Journal/nar/53/11/10.1093_nar_gkaf485/1/gkaf485_supplemental_files.zip?Expires=1753205889&Signature=rVSjWwEmbwT0fSZOBEnmvh76IRhXdvs~uC46NZ0r-DhTdgJRt1pgRAlKm~8cTkOfNNUVhGLyVFuh58Ob3v0~et0ayTCgZjPJSTtxr3XfEcULbQsuw2ZR1OWNRLNfvtyu2QJg21MNvrJtMOEw8OftiI8Ri96ZR1DY0~zUdgkDQ~JoHwG4W6uPh83KBT2WgZCg6tlmDwMtAdmM~QEGRVQCMxCDe0SrOS9umcTOwelAVEpKa~bXCoSKZOnzhi2~FjYCuS7pEnMLP6I1dMVoUa~ah2-cHd4KC9Y56D-xcTAH40GBllbH2jKXMGSfeka57rhH~f9tKumiue1dk-IRgtpGig__&Key-Pair-Id=APKAIE5G5CRDK6RD3PGA) **Figure 17.** 10% dPAGE analysis of the click-ligating activity of various CDLz2 mutants after mutation of nucleotides in J1/2 and L2. Reaction time: 24 h, lig: ligated product, un-lig: un-ligated product, Lig%: ligation percentage, M: marker.

**Uncropped gel images**


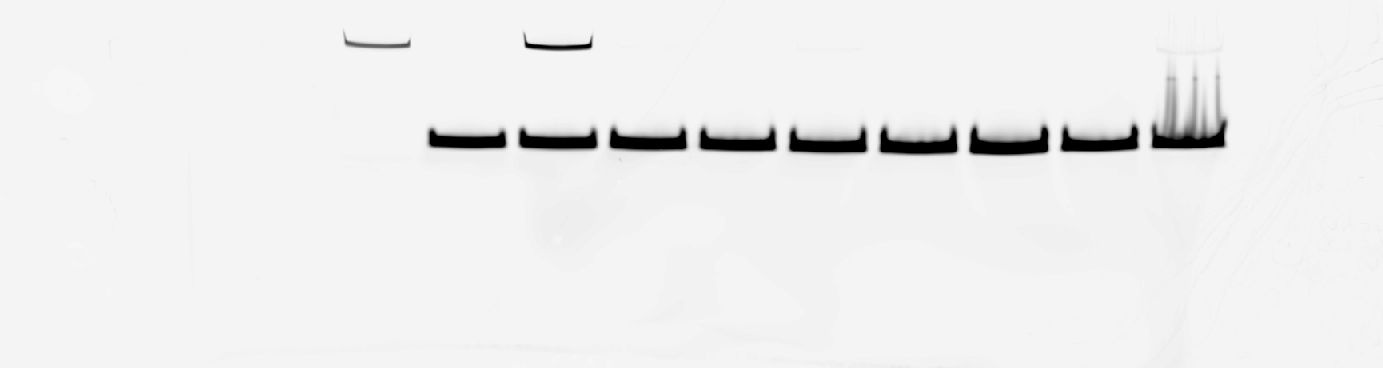
Uncropped dPAGE gel for Figure 2d. The red box indicates the region presented in Figure 2d.

Supplementary Figure 2d


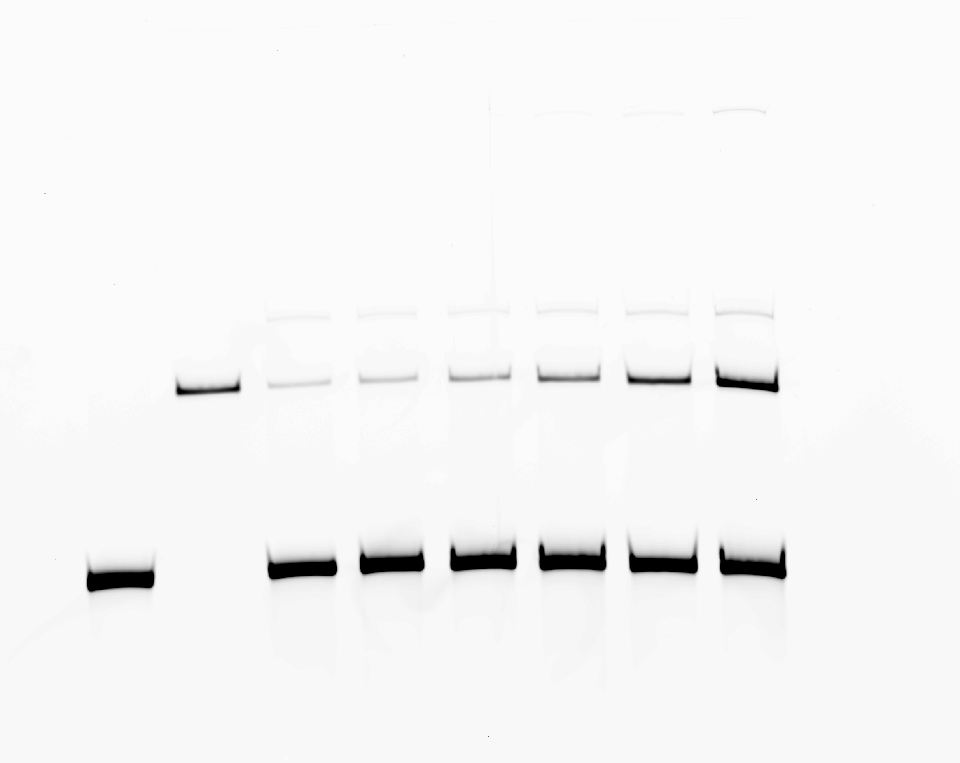


Figure 6a

Uncropped dPAGE gel for Figure 6a. The red box indicates the region presented in Figure 6a.

**
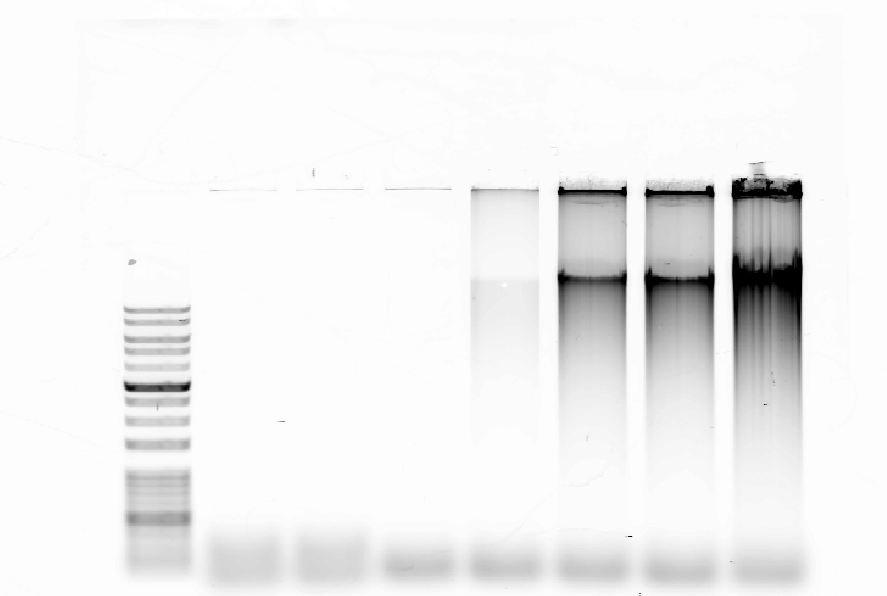
**

Figure 6b

Uncropped dPAGE gel for Figure 6b. The red box indicates the region presented in Figure 6b.


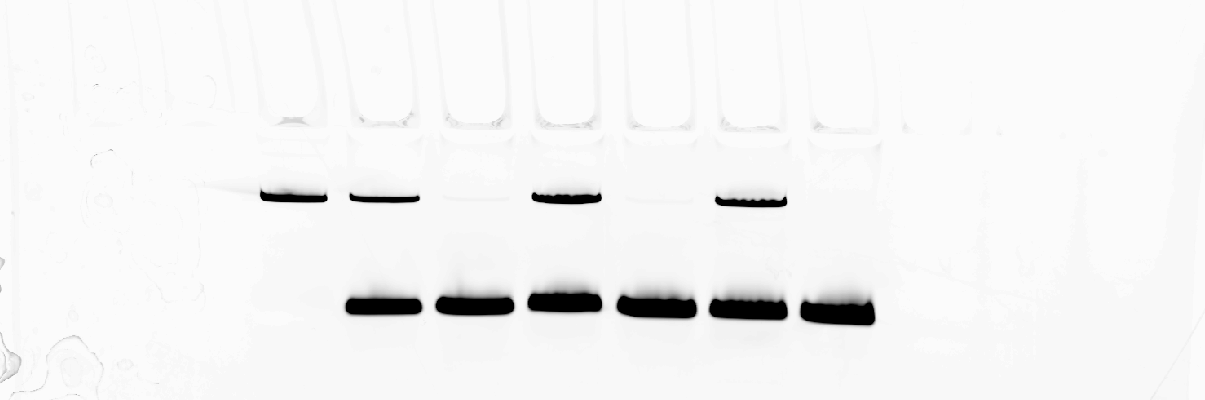
 Uncropped dPAGE gel for Supplementary Figure 2. The red box indicates the region presented in Supplementary Figure 2.

Supplementary Figure 2


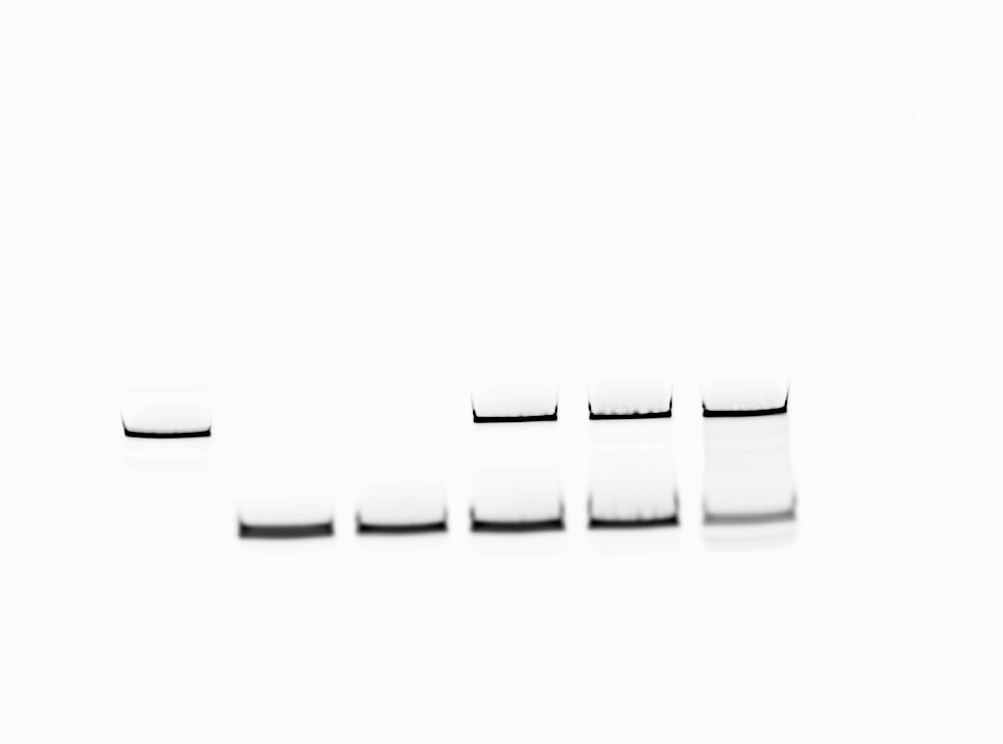


Supplementary Figure 3

Uncropped dPAGE gel for Supplementary Figure 3. The red box indicates the region presented in Supplementary Figure 3.


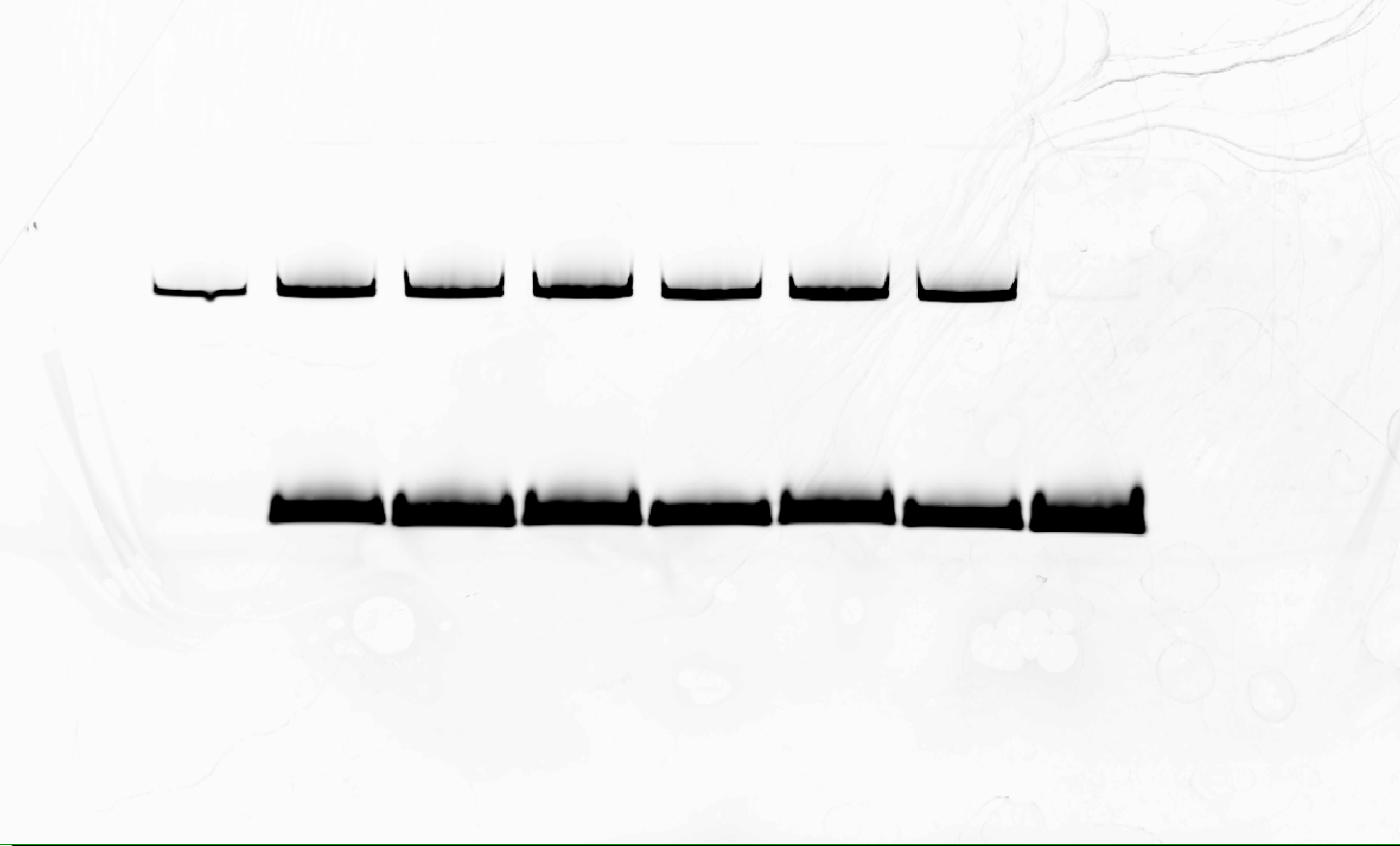
 Uncropped dPAGE gel for Supplementary Figure 4. The red box indicates the region presented in Supplementary Figure 4.

Supplementary Figure 4


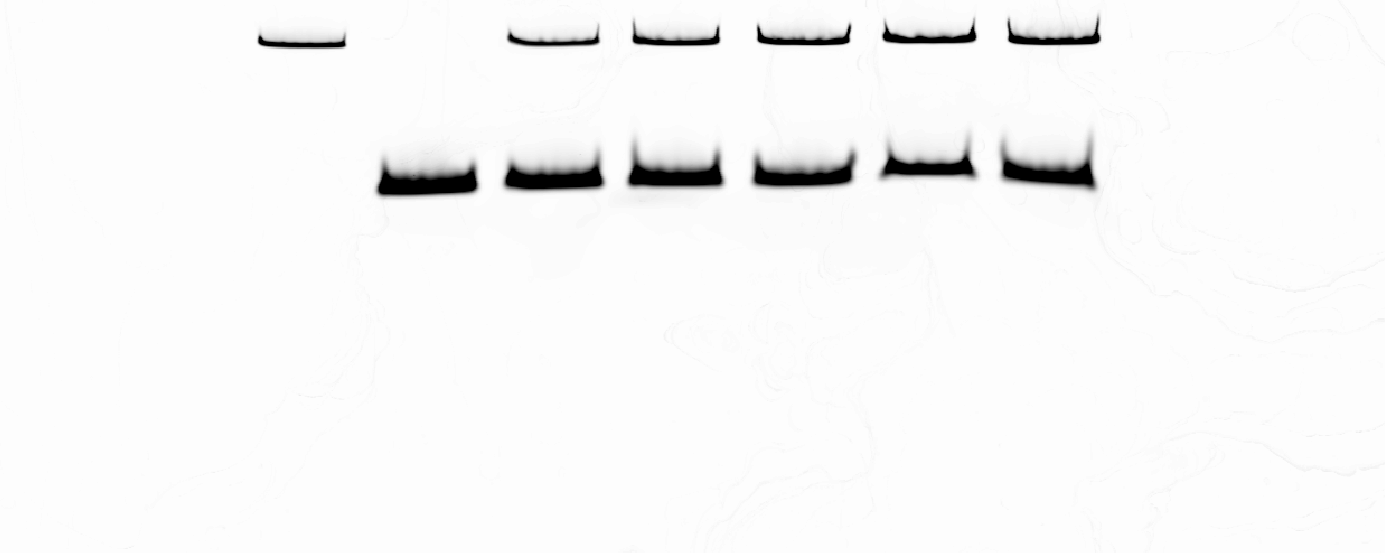
 Uncropped dPAGE gel for Supplementary Figure 5. The red box indicates the region presented in Supplementary Figure 5.

Supplementary Figure 5


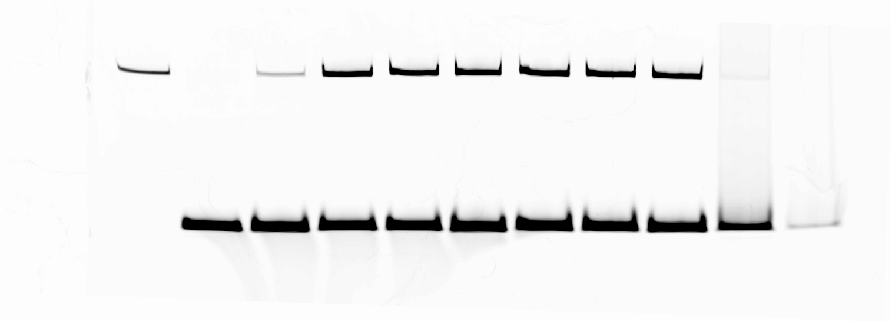
 Uncropped dPAGE gel for Supplementary Figure 6. The red box indicates the region presented in Supplementary Figure 6.

Supplementary Figure 6


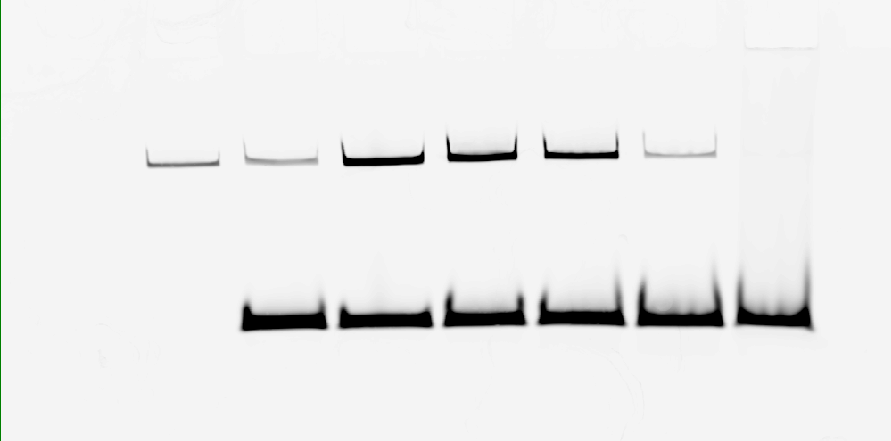


Supplementary Figure 7

Uncropped dPAGE gel for Supplementary Figure 7. The red box indicates the region presented in Supplementary Figure 7.


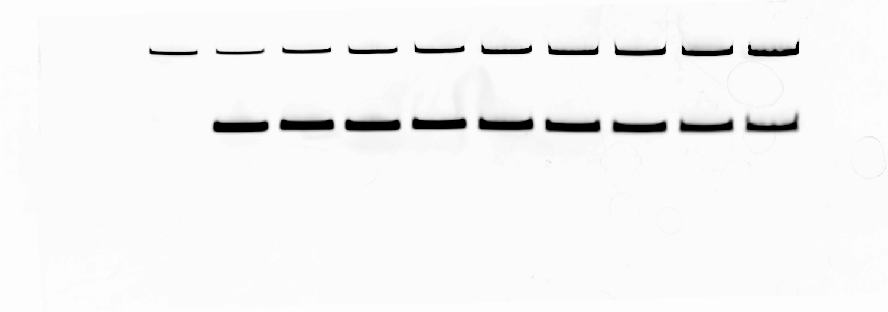
 Uncropped dPAGE gel for Supplementary Figure 8. The red box indicates the region presented in Supplementary Figure 8.

Supplementary Figure 8


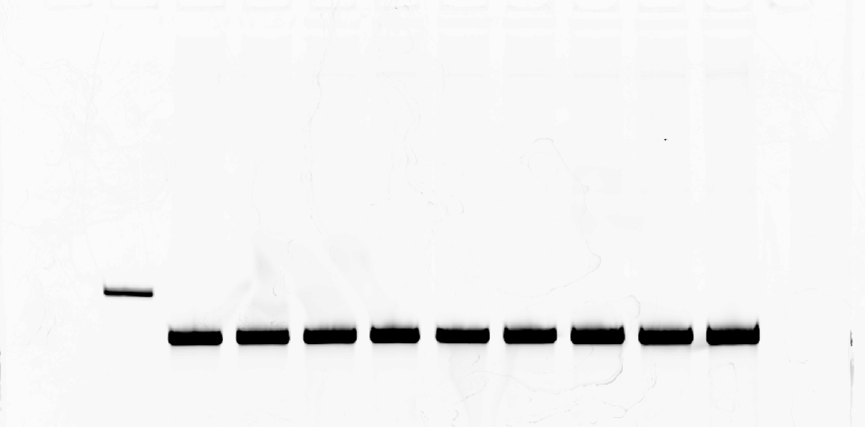
 Uncropped dPAGE gel for Supplementary Figure 9. The red box indicates the region presented in Supplementary Figure 9.

Supplementary Figure 9


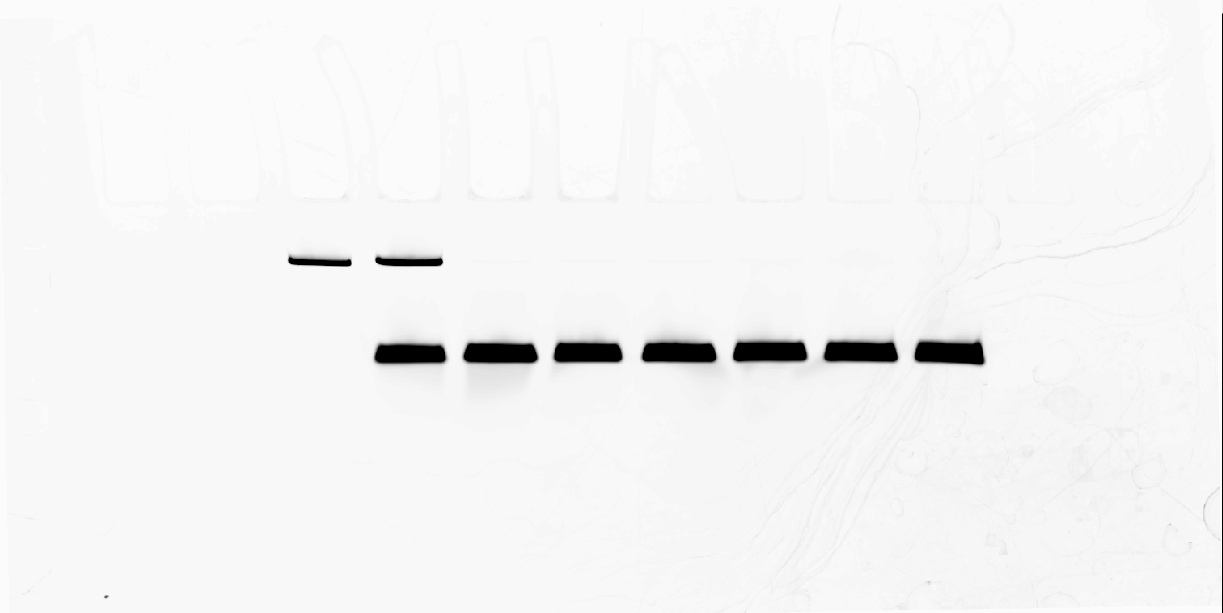


Supplementary Figure 10

Uncropped dPAGE gel for Supplementary Figure 10. The red box indicates the region presented in Supplementary Figure 10.


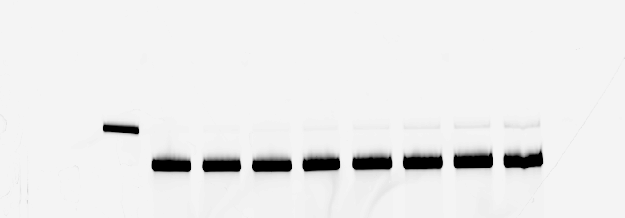
 Uncropped dPAGE gel for Supplementary Figure 11. The red box indicates the region presented in Supplementary Figure 11.

Supplementary Figure 11


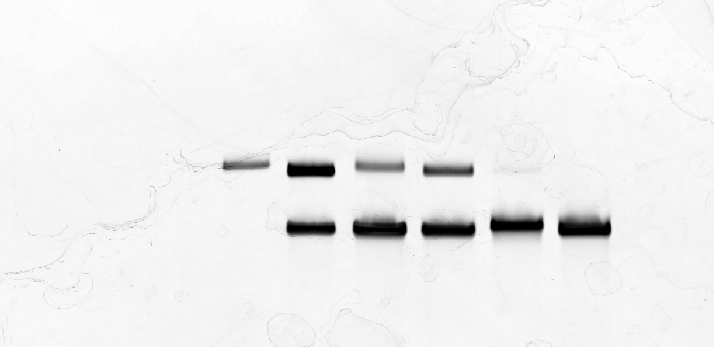


Supplementary Figure 12

Uncropped dPAGE gel for Supplementary Figure 12. The red box indicates the region presented in Supplementary Figure 12.


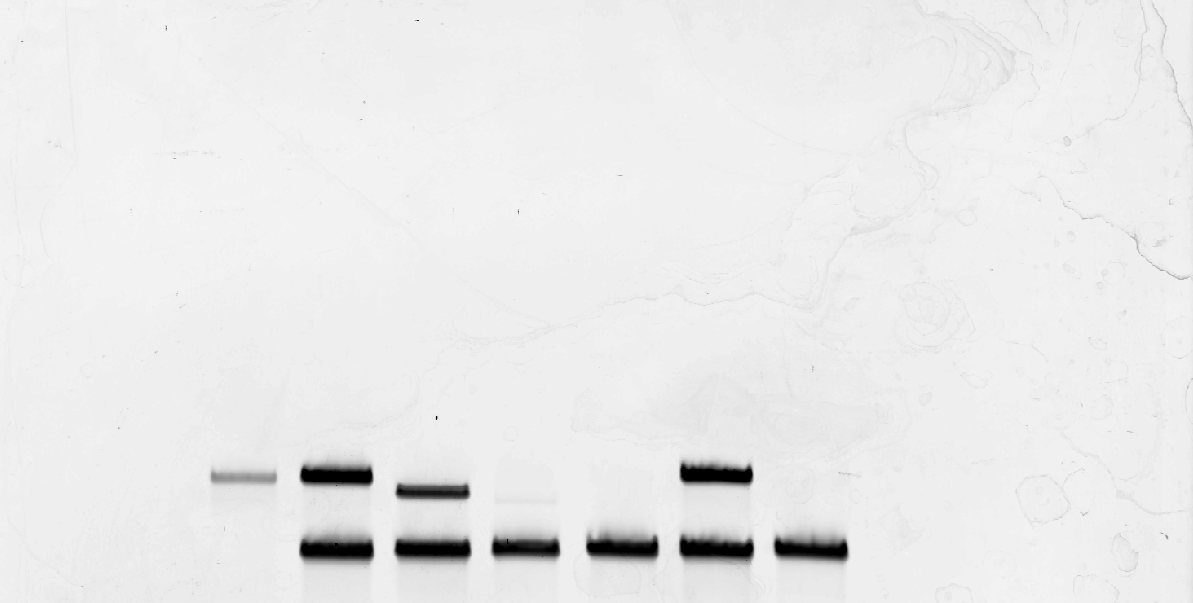
 Uncropped dPAGE gel for Supplementary Figure13. The red box indicates the region presented in Supplementary Figure 13.

Supplementary Figure 13


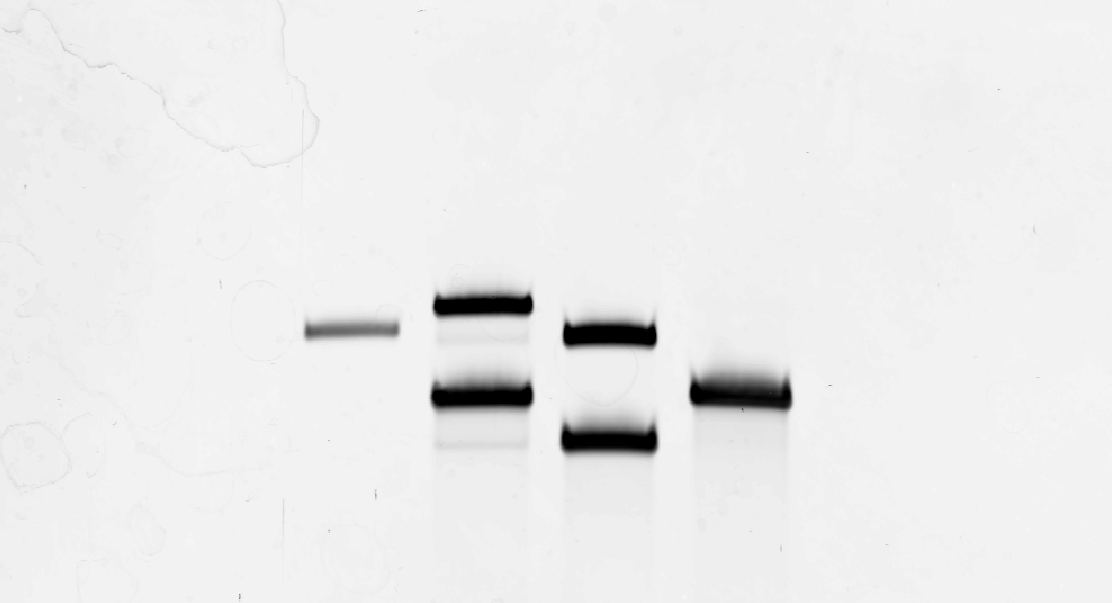


Supplementary Figure 14

Uncropped dPAGE gel for Supplementary Figure 14. The red box indicates the region presented in Supplementary Figure 14.


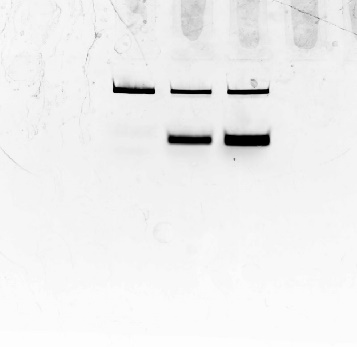

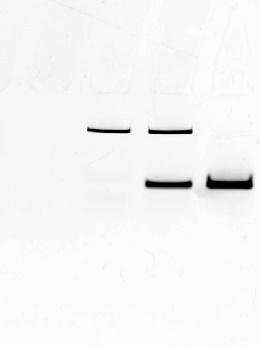

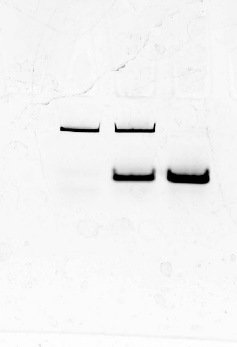

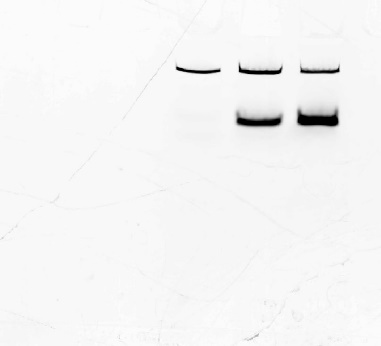


Supplementary Figure 16 top


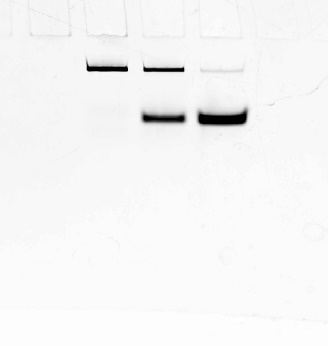

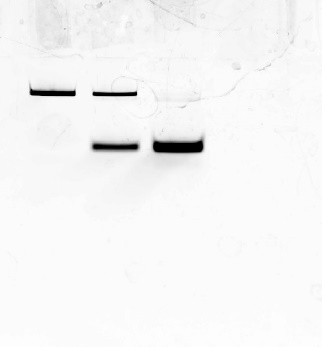

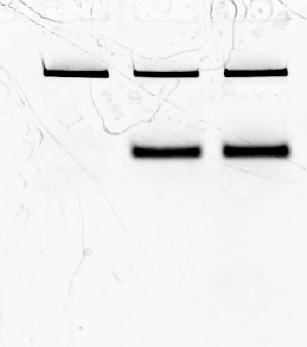


Supplementary Figure 16 bottom

Uncropped dPAGE gel for Supplementary Figure 16. The red box indicates the region presented in Supplementary Figure 16.


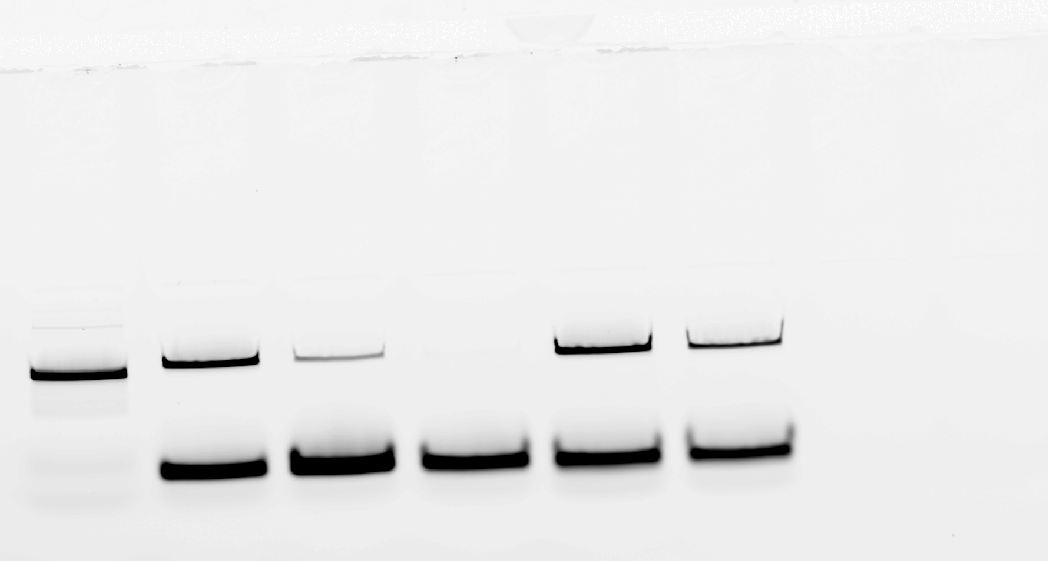

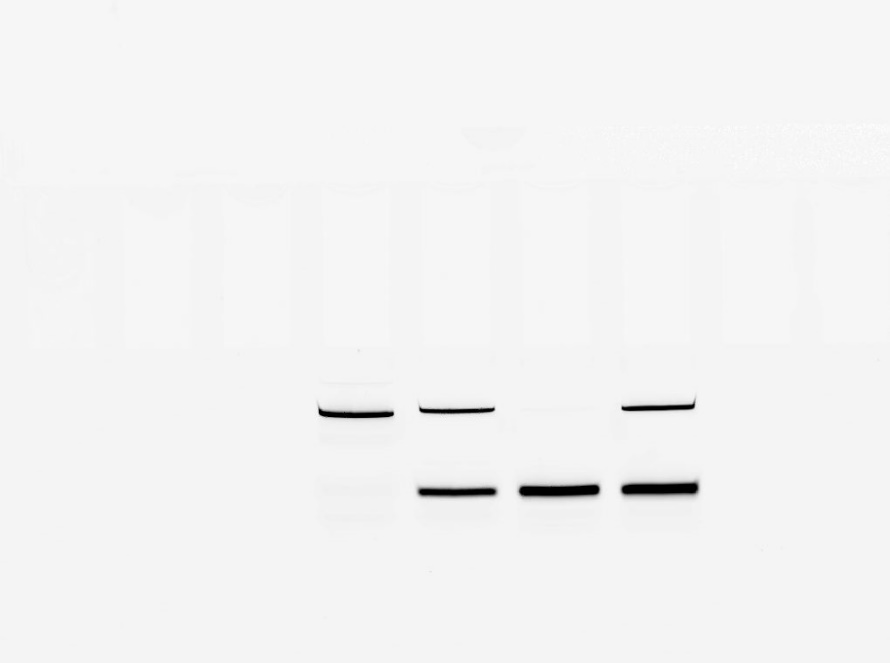


Supplementary Figure 17 right

Supplementary Figure 17 left

Uncropped dPAGE gel for Supplementary Figure 17. The red box indicates the region presented in Supplementary Figure 17.
